# Methodologies for the synthesis of quaternary carbon centers via hydroalkylation of unactivated olefins: twenty years of advances

**DOI:** 10.3762/bjoc.17.112

**Published:** 2021-07-07

**Authors:** Thiago S Silva, Fernando Coelho

**Affiliations:** 1 Laboratory of Synthesis of Natural Products and Drugs, Department of Organic Chemistry, Chemistry Institute, University of Campinas, PO Box 6154 - 13083-970, Campinas – SP, Brazil

**Keywords:** hydroalkylation, hydrogen atom transfer, quaternary carbon center, radical addition, unactivated olefins

## Abstract

Olefin double-bond functionalization has been established as an excellent strategy for the construction of elaborate molecules. In particular, the hydroalkylation of olefins represents a straightforward strategy for the synthesis of new C(sp^3^)–C(sp^3^) bonds, with concomitant formation of challenging quaternary carbon centers. In the last 20 years, numerous hydroalkylation methodologies have emerged that have explored the diverse reactivity patterns of the olefin double bond. This review presents examples of olefins acting as electrophilic partners when coordinated with electrophilic transition-metal complexes or, in more recent approaches, when used as precursors of nucleophilic radical species in metal hydride hydrogen atom transfer reactions. This unique reactivity, combined with the wide availability of olefins as starting materials and the success reported in the construction of all-carbon C(sp^3^) quaternary centers, makes hydroalkylation reactions an ideal platform for the synthesis of molecules with increased molecular complexity.

## Introduction

**Figure 1:**
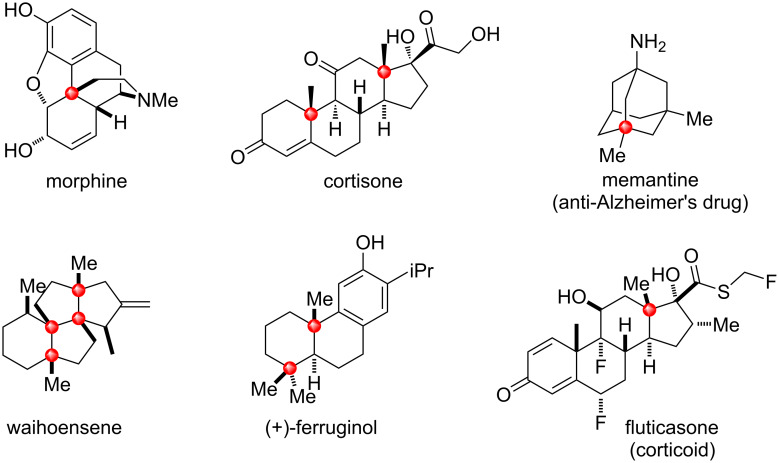
Some examples of natural products and drugs containing quaternary carbon centers.

Natural product structures remain some of the main sources of inspiration for the synthesis of new bioactive compounds [1–2]. A careful view of the carbon backbone of these natural molecules reveals their high molecular complexity [3–5], which can be described by the presence of multiple stereogenic centers in the same molecule, a substantial fraction of sp^3^ hybridized carbons (Fsp^3^) [6], and the presence of all-carbon quaternary centers (Figure 1). The all-carbon quaternary center motif represents a challenge in modern organic synthesis due to the inherent steric issues associated with the formation of these particular C(sp^3^)–C(sp^3^) bonds [7–13]. A glimpse of the relevance of this moiety was given by Overman in 2014 [14], who stated that about 12% of the 200 most predicted drugs in the U.S.A. in 2011 had at least one quaternary carbon center, but this center was not synthetically constructed in any of them.

Interest is currently growing in increasing the molecular complexity of drugs in drug discovery programs, aiming at molecules with better selectivity for biological targets [6,15–16]. One ideal way to achieve more complex molecular architectures is by hydroalkylation of olefins, a reaction that forms new C(sp^3^)–C(sp^3^) and C(sp^3^)–H bonds at the expense of a double bond by increasing the Fsp^3^ with the concomitant formation of carbon quaternary centers.

Olefins are desirable starting materials, as they are usually low-cost and stable chemicals that are readily available from natural sources (e.g., terpenes [3–5]) or from industrial processes (e.g., styrene derivatives [17]) that produce hundreds of tons for polymer synthesis. The goal of this review is to demonstrate the utility of olefins as starting materials in the synthesis of all-carbon quaternary centers through hydroalkylation reactions. Only the hydroalkylation of unactivated olefins as substrates will be reviewed, so electron-deficient olefins like Michael acceptors and even conjugate dienes will not be included in this review. Some examples of hydroallylation reactions have been included since these reactions, like hydroalkylation reactions, also form a new C(sp^3^)–C(sp^3^) bond at the expense of the olefin double bond.

## Review

### Olefin activation by transition metals

**Scheme 1:**
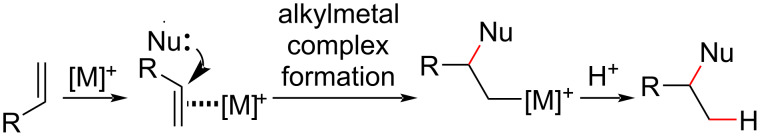
Simplified mechanism for olefin hydrofunctionalization using an electrophilic transition metal as a catalyst.

Transition metals can act as π-acid catalysts in olefin double bond activation, as they weaken the alkene double bond through coordination to allow a nucleophilic attack on the sp^2^ carbon to form an alkylmetal intermediate [18–21]. In the presence of a Brønsted acid source, the alkylmetal bond can be transformed into a C–H bond to afford a hydrofunctionalized olefin product (Scheme 1) [22].

The relevance of the formation of new C(sp^3^)–C(sp^3^) bonds has led to great efforts to enable the use of carbon nucleophiles, along with transition-metal catalysis, in olefin alkylation reactions [18–21]. Apart from the early examples of olefin functionalization with transition metals, such as the well-known ethylene oxidation catalyzed by Pd(II) (the Wacker reaction) [23–24], the use of carbon nucleophiles in olefin hydroalkylation reactions using catalytic amounts of a transition metal was reported for the first time in 2001, about 42 years after the introduction of the Wacker reaction, in the seminal work by Widenhoefer [25]. Since then, more sophisticated strategies have been developed, and systems other than Pd(II)-catalytic types have also been explored.

#### Pd(II)-catalyzed hydroalkylation reactions

The σ-alkylpalladium species formed after a carbon nucleophilic attack on an alkene double bond (Scheme 1) have a marked tendency to undergo a hydride β-elimination process that leads to oxidative coupling products instead of reductive ones. Their formation also leads to the generation of Pd(0) species that require the presence of an external oxidant to regenerate the active Pd(II) complex, as well as an additional hydrogenation step, if the hydrofunctionalized product is desired [18]. This particularity hampered the development of more general catalytic hydrofunctionalization methodologies until Widenhoefer’s work in 2001 [25].

**Scheme 2:**
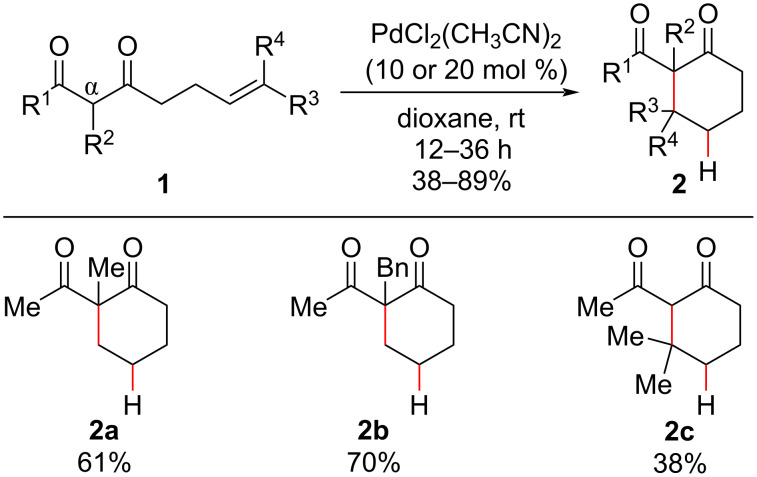
Selected examples of quaternary carbon centers formed by the intramolecular hydroalkylation of β-diketones using a Pd(II) source (Widenhoefer (2001) [25]).

Based on previous works that had demonstrated the addition of stabilized carbanions [26–27] and enol silyl ethers [28] to olefins under stoichiometric Pd catalysis, the authors reported the use of active methylene compounds as suitable nucleophiles in intramolecular hydroalkylation reactions (Scheme 2) [25]. In the presence of PdCl_2_(CH_3_CN)_2_, the hydroalkylation of β-diketones **1** gave only 6-*endo*-*trig* cyclization products **2** in moderate to good yields (Scheme 2). The methodology proved suitable for the synthesis of quaternary centers, although only moderate yields were observed in these cyclizations, and a significant decrease in reactivity was observed when the geminal disubstituted pendant olefin **2c** was the cyclization substrate (38% yield) compared with the use of α-substituted β-diketones **2a** and **2b** (61 and 70% yield, respectively). Notably, the unsaturated carbocycles expected from palladium β-hydride elimination were not observed, indicating that an oxidant was not required in the reaction medium to regenerate the Pd(II) species.

**Scheme 3:**
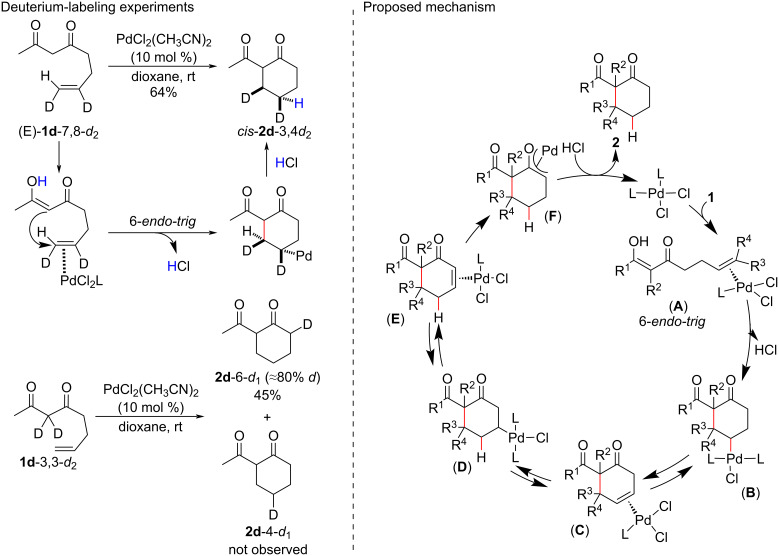
Control experiments and the proposed mechanism for the Pd(II)-catalyzed intermolecular hydroalkylation of β-diketones (Widenhoefer (2005) [29]).

Later, deuterium-labeling experiments helped to elucidate the operative mechanism of the hydroalkylation (Scheme 3) [29]. The outer-sphere attack of the enolic carbon on the complexed metal–olefin double bond was evidenced by the cyclization of (*E*)-**1d-**7,8-*d*_2_, which furnished exclusively the compound *cis*-**2d**-3,4-*d*_2_ after the protonolysis of the Pd(II) intermediate, with retention of the configuration. When **1d**-3,3-*d*_2_ was used in the presence of the Pd(II) catalyst, only the isotopomer **2d**-6-*d*_1_ was obtained, indicating the migration of the Pd(II) alkyl complex from the site of the cyclization C(4) to the α-carbonyl position and pointing to the protonation of a Pd(II) enolate intermediate by a proton source (HCl) generated at the cyclization step.

The catalytic cycle begins with the enolic carbon of **A** attacking the complexed metal–olefin double bond in a turnover-limiting 6-*endo*-*trig* cyclization step (Scheme 3). The formed alkylpalladium(II) intermediate (**B**) then undergoes a sequence of reversible hydride β-eliminations [30] until the formation of the Pd(II) enolate (**F**), which, after protonation, irreversibly furnishes the product **2** and regenerates the Pd(II) catalyst. The lack of the usually kinetically favored 5-*exo*-*trig*-cyclization products was attributed to the better overlap between the π-orbital of the enolic carbon atom and the π*-orbital of the complexed olefin terminal carbon. This suggests that, in these cases, the 6-*endo*-*trig* cyclizations are under kinetic control.

**Scheme 4:**
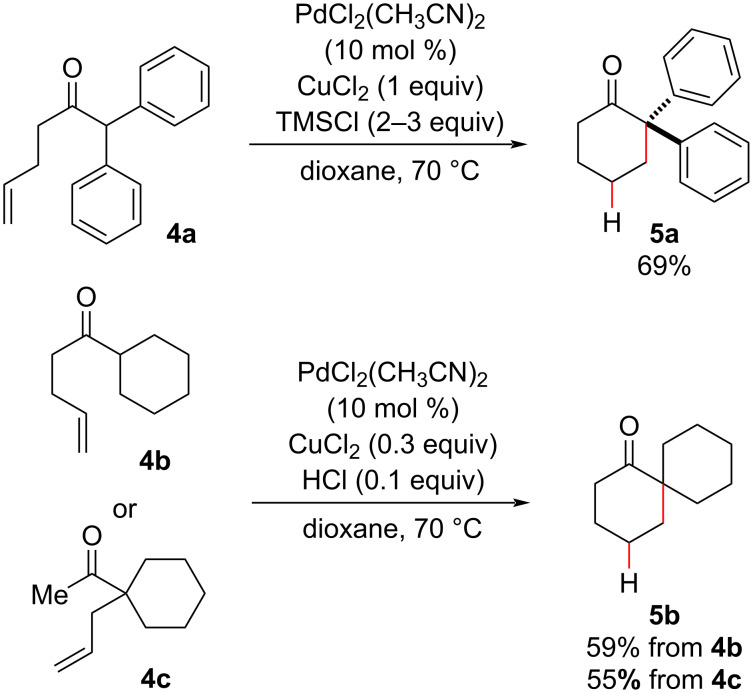
Intermolecular olefin hydroalkylation of less reactive ketones under Pd(II) catalysis using HCl as an additive for the synthesis of quaternary carbon centers (Widenhoefer (2003) [32]).

The substrate generality in the methodology developed by Widenhoefer [25,29] relied heavily on the acidity of the carbonyl substrate, and this hampered the use of less enolizable substrates, like alkenyl β-ketoesters. The same group addressed this limitation by proposing the use of trimethylsilyl chloride (TMSCl) as an additive in the intramolecular hydroalkylation reactions of less activated substrates [31]. Later studies demonstrated that the true reason for the reaction success was not the putative in situ formation of silyl enol ethers but the HCl formation due to TMSCl hydrolysis. The HCl catalyzed the formation of the enolic form responsible for the nucleophilic attack on the metal-complexed olefin (see Scheme 3, intermediate **A**). Taking advantage of HCl as an additive (or generated in situ by silyl chloride hydrolysis), Widenhoefer described the intramolecular hydroalkylation of even less reactive alkenyl ketones **4** under Pd(II) catalysis, with minor adjustments from the previous conditions (Scheme 2) [25], such as conducting the reaction at a higher temperature and adding a copper salt to reoxidize possible Pd(0) species produced in the cyclization process [32]. Two compounds containing quaternary centers were synthetized in moderate yields (Scheme 4), and notably, hydroalkylation products were produced from α-aryl ketones, from more acidic substrates like **4a**, and from α-alkyl ketones, including the less activated methyl ketone **4c**.

Examples of intermolecular hydroalkylations of olefins using Pd were much scarcer than their intramolecular counterparts, and so therefore are examples of quaternary centers generated by this means. The tendency of σ-alkylpalladium complexes to undergo a β-hydride elimination process is the major obstacle associated with the development of successful methodologies to promote intermolecular hydroalkylation of unactivated olefins [33–34].

**Scheme 5:**
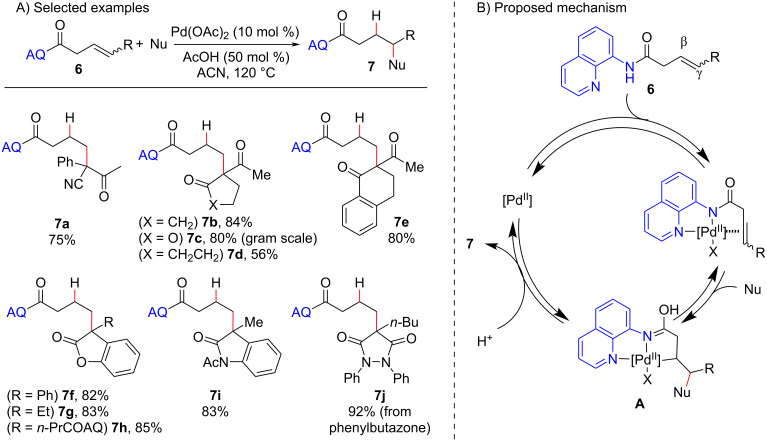
A) Selected examples of Pd(II)-mediated quaternary carbon center synthesis by intermolecular hydroalkylation of olefins. B) Proposed mechanism (Engle (2016) [35]).

The observed competition between protodemetalation and β-hydride elimination steps was elegantly overcome by Engle and co-workers through the use of olefins tethered to the directing group 8-aminoquinoline (AQ) amide. This amide trapped the carbometalated intermediate generated after the nucleophilic addition as a stable bis-5,5-palladacycle (**A**), thereby preventing the β-hydride elimination step and allowing its further reaction with a proton to yield intermolecular hydroalkylation products (Scheme 5) [35]. In that work, the authors considerably expanded the scope of suitable nucleophiles beyond the usually employed dicarbonyl compounds to cyanoacetates, nitroacetates, fluoroacetates, lactones, lactams, and aromatic and heteroaromatic carbonyl compounds. The reactions were carried out at higher temperatures (120 °C) than used in the previous protocols involving more reactive substrates (Scheme 2 and Scheme 4), and an external proton source (acetic acid) was required to furnish the hydroalkylated products **7** (Scheme 5A). The quaternary carbon centers were synthetized in moderate to excellent yields by this methodology, and its potential use in late-stage functionalization was further demonstrated by the hydroalkylation of the anti-inflammatory drug phenylbutazone (**7j**).

#### Gold(I)/gold(III) and silver(I) catalysis in olefin hydroalkylation

The early limitations associated with Pd catalysis in olefin hydroalkylation reactions prompted the search for alternative metals for olefin activation. The use of metals in which the protodemetalation step of the σ-alkylmetal complex occurs preferentially to the β-hydride elimination arose as an alternative for the development of new methodologies. During the early 2000s, several synthetic methodologies flourished that employed gold catalysis to activate unsaturated bonds, and examples of olefin hydroalkylation, hydroarylation, hydrofluorination, hydroamination, and aminoheteroarylation, among others, can be found in the literature [36–37].

**Scheme 6:**
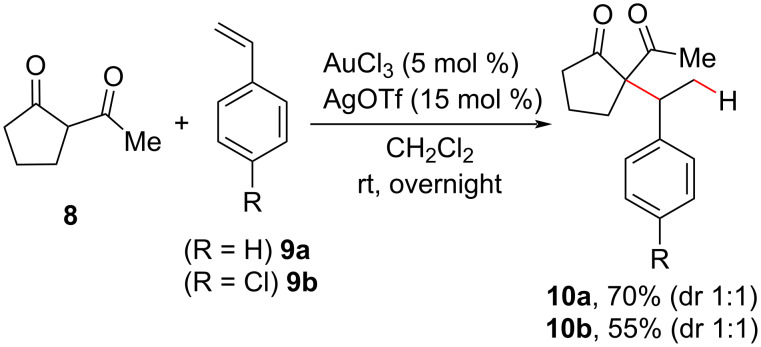
Selected examples of quaternary carbon center synthesis by gold(III) catalysis. This is the first report of a gold catalysis in the hydroalkylation of olefins (Li (2004) [38]).

The first example of an olefin hydroalkylation promoted by gold catalysis appeared in 2004 in the work by Li [38], who used a catalytic system of AuCl_3_/AgOTf to promote the addition of active methylene compounds like acetylcyclopentanone **8** to more reactive alkenes, such as styrenes **9**. Two examples were reported by the authors for the synthesis of quaternary carbon centers without the necessity of changing the previously optimized mild reaction conditions (Scheme 6). Only traces of **10** were observed in the absence of one of the metal salts; this excluded the participation of the silver salt in the olefin activation but highlighted its usual role in the activation of the gold catalyst, namely halide abstraction, to form a gold complex with a less coordinating ligand that serves as the active catalyst [39].

**Scheme 7:**
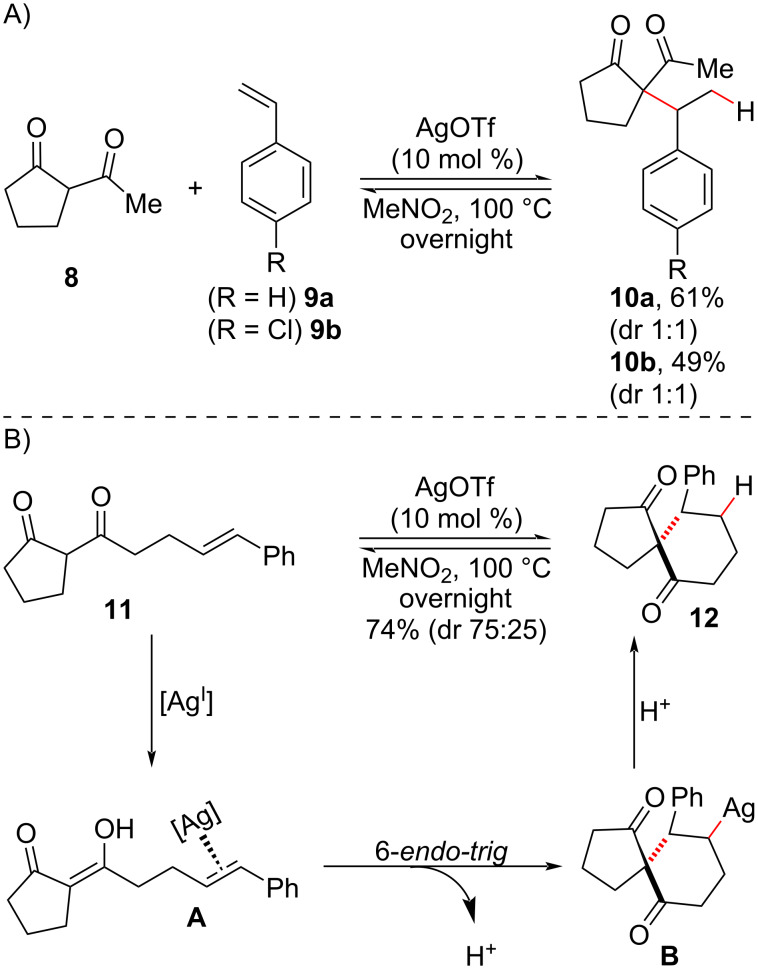
Selected examples of inter- (A) and intramolecular (B) olefin hydroalkylations promoted by a silver(I) salt (Li (2005) [40]).

Following their initial work, the Li group investigated the use of silver triflate as the sole catalyst in the olefin hydroalkylation [40]. In their previous work, they had reported that neither the gold(III) salt AuCl_3_ nor the silver additive AgOTf was able to catalyze the reaction alone under the screened conditions. At elevated temperatures, however, the authors found that inter- and intramolecular olefin hydroalkylations occurred using AgOTf alone as the catalyst, leading to Markovnikov addition products **10** and **12** (Scheme 7A). In both cases, a low diastereoselective control was observed. Control experiments revealed that, under these reaction conditions, the hydroalkylation was reversible (Scheme 7B), indicating the participation of the silver salt in the C(sp^3^)–C(sp^3^)-bond cleavage.

**Scheme 8:**
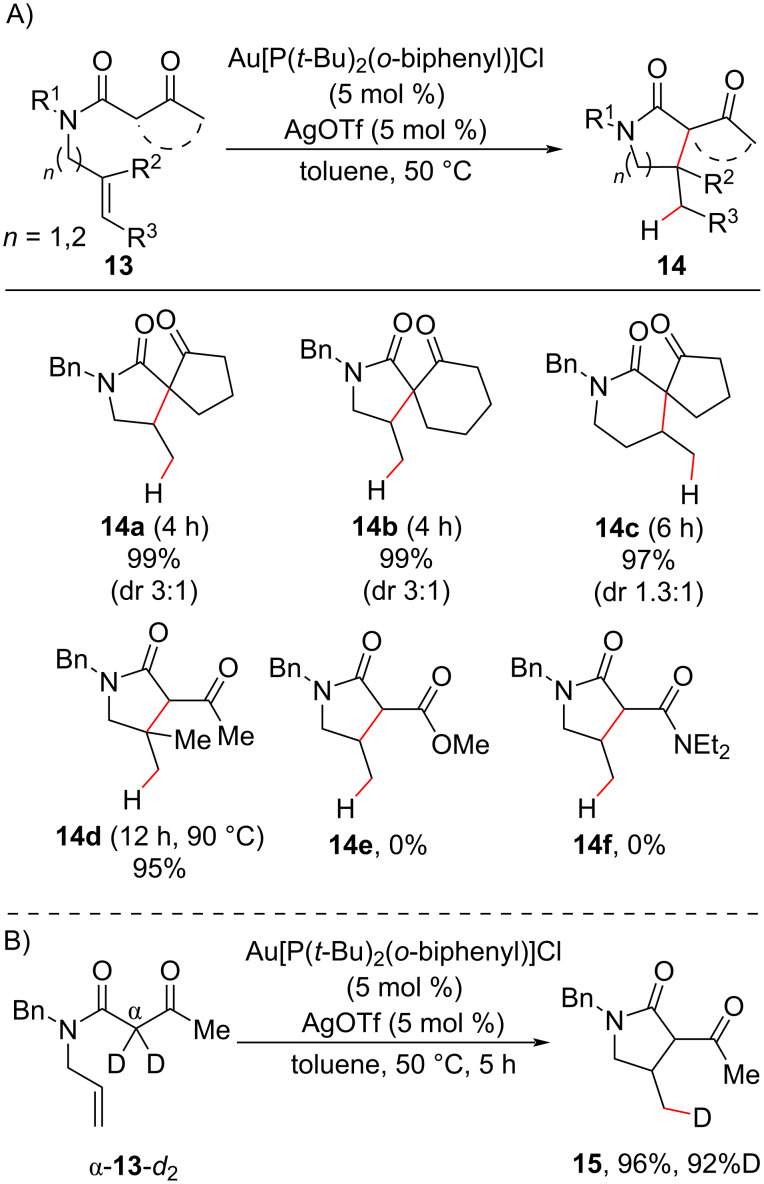
A) Intermolecular hydroalkylation of *N*-alkenyl β-ketoamides under Au(I) catalysis in the synthesis of quaternary carbon centers. B) Deuterium-labeled experiment (Che (2007) [41]).

Cationic gold(I) complexes are also suitable catalysts for olefin functionalization, and their use has become more popular than gold(III) catalysis [39]. In 2007, Che and Zhou reported the olefin intramolecular hydroalkylation of *N*-alkenyl β-ketoamides **13** using the gold(I)–phosphine complex Au[P(*t*-Bu)_2_(*o*-biphenyl)]Cl as a catalyst (Scheme 8) [41]. Under mild conditions, functionalized five and six-membered lactams **14** were synthetized in excellent yields (90–99%), including those of the lactams in which a quaternary center was constructed. Only *exo*-*trig* cyclization products were obtained, and substrates containing a disubstituted olefin carbon were also effective (Scheme 8A, **14e**), although they required higher temperatures and long reaction times. Substrates containing a β-ester or a β-amide instead of a β-keto group failed to deliver the cyclization products (Scheme 8A, compounds **14e** and **14f**), probably because of the less enolizable character of these compounds. Deuterium incorporation at the olefin terminal carbon was obtained when the deuterated substrate α-**13**-*d*_2_ was subjected to the reaction conditions, indicating the participation of an internal proton source in the putative protodemetalation step (Scheme 8B).

**Scheme 9:**
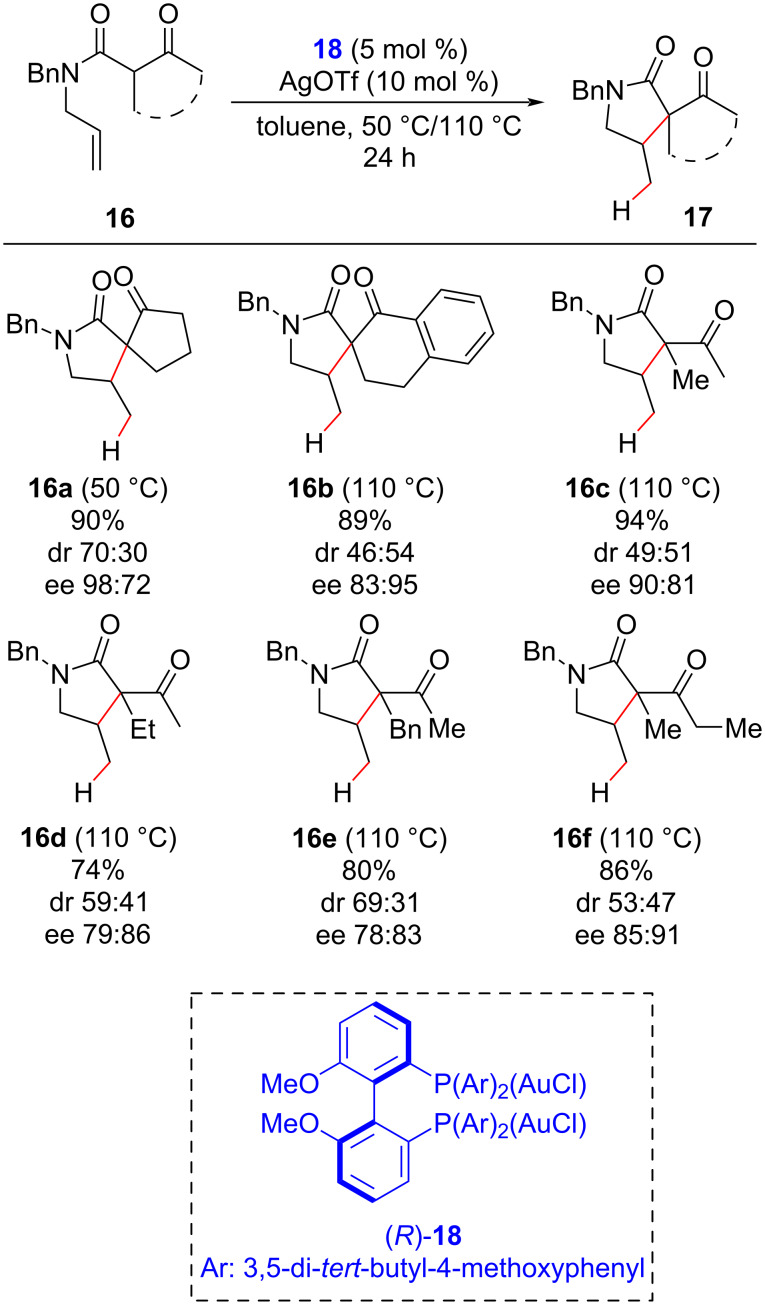
Asymmetric pyrrolidine synthesis through intramolecular hydroalkylation of α-substituted *N*-alkenyl β-ketoamides by chiral gold catalysis (Gandon (2014) [42]).

An asymmetric version of this reaction was developed in 2014 by the Gandon group [42], who employed the chiral bis(phosphine)digold(I) complex **18** as a pre-catalyst in combination with silver triflate as an activator (Scheme 9). They obtained lactams **17** by cyclization of α-substituted *N*-alkenyl β-ketoamides **16**, in yields ranging from 74% to 94%, in excellent enantiomeric excess (up to 98% ee), and in modest to poor diastereoisomeric ratios (dr up to 70:30).

**Scheme 10:**
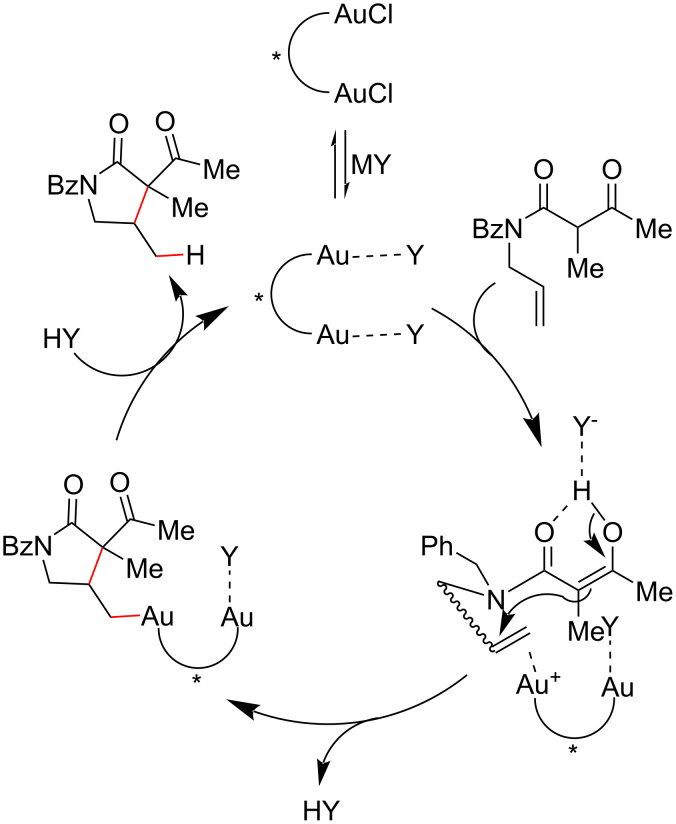
Proposed mechanism for the chiral gold(I) complex promotion of the intermolecular olefin hydroalkylation of an *N*-alkenyl β-ketoamide (Gandon (2014) [42]).

The authors observed that the nature of the activator had a substantial influence on the reaction enantio- and diastereoselectivity, suggesting that the coordination and proton-shuttle ability of the anionic counterion were the main factors responsible for the observed effects. The proposed mechanism (Scheme 10) begins with the activation of the gold(I) pre-catalyst by the exchange of a chloride anion with a non-coordinated one (Y^−^) to generate the active gold(I) catalyst that coordinates with the olefin. An intramolecular attack of the enol form of **16** on the activated double bond then leads to the formation of an alkyl–gold complex that, after protonolysis, furnishes the hydroalkylated product.

**Scheme 11:**
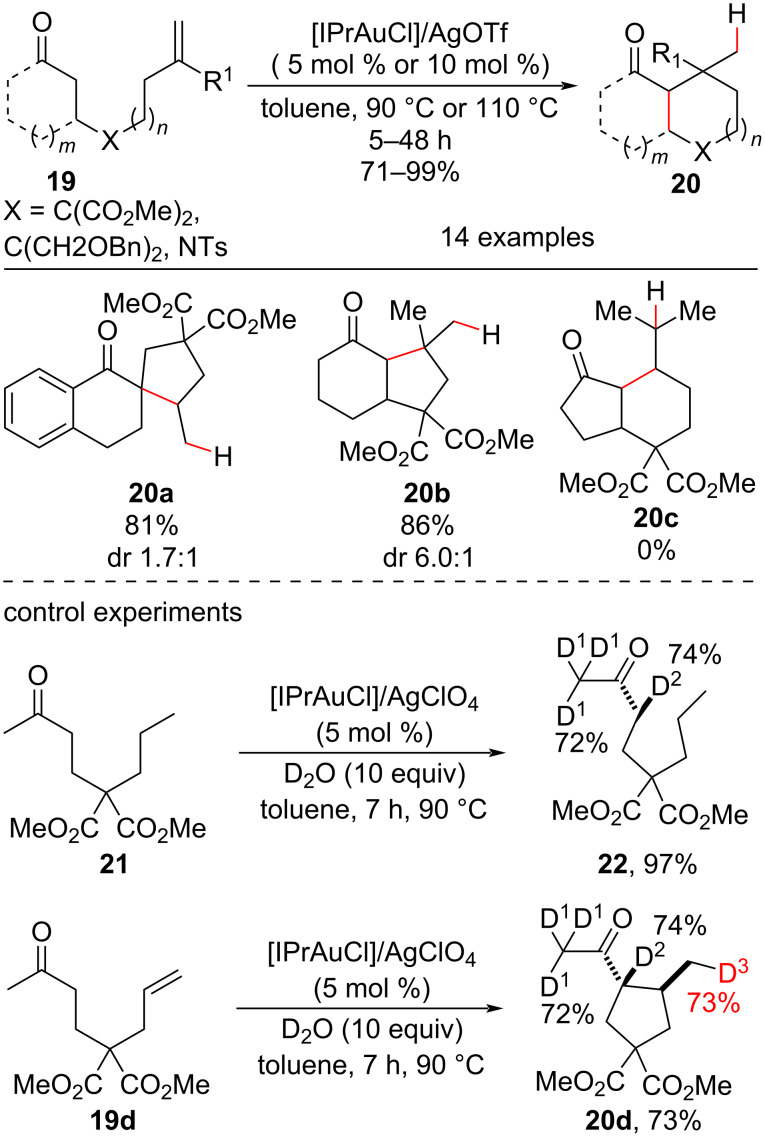
Selected examples of carbon quaternary center synthesis by gold and evidence of catalytic system participation in the ketone enolization (Che (2011) [44]).

The ability of gold(I) complexes to promote the enolization of carbonyl compounds [43] allowed the use of simple ketones as substrates in the intramolecular hydroalkylation of olefins. As discussed before, these are problematic substrates because of the low equilibrium constant between the keto and enol forms. Che’s group used [IPrAuCl]/AgClO_4_ as a catalyst system and reported the hydroalkylation of tethered alkenyl ketones **19** (Scheme 11) to obtain functionalized cyclic compounds (Scheme 11) [44]. Among the products, two examples of quaternary carbon center syntheses (**20a** and **20b**) were both obtained in good yields. Products from the cyclization of internal alkenes, such as **19c**, were not generated by this methodology. Additional experiments in the presence of deuterated water corroborated the authors’ proposal of an involvement of gold catalysis in the enolization of the substrates (a hydrogen–deuterium [H–D] exchange in saturated substrate **21** was observed at the α-carbonyl positions only in the presence of the metal catalysis). The authors also observed a H–D exchange at the terminal carbon of **20d**, which supported the proposal of an *exo*-*trig* cyclization followed by a protodemetalation step.

**Scheme 12:**
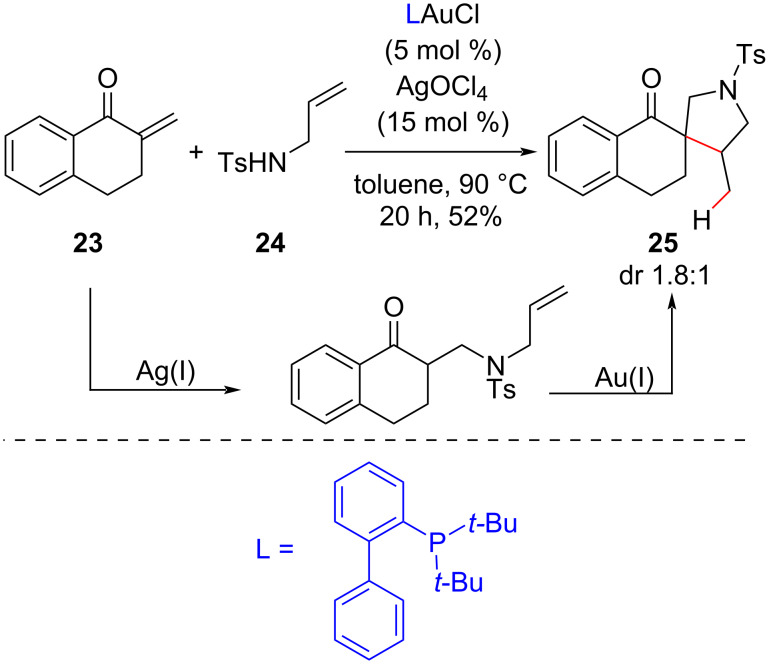
Synthesis of a spiro compound via an aza*-*Michael addition/olefin hydroalkylation cascade promoted by a Au(I)/Ag(I) catalytic system (Che (2011) [45]).

Subsequently, the same group reported a cascade aza-Michael addition/olefin hydroalkylation reaction between *N*-tosylallylamines and α,β-unsaturated ketones using a catalytic system of a gold(I) complex and a silver salt [45]. The spiro compound **25**, which was obtained in moderate yield and with poor diastereoselectivity after a 20 h reaction, was the only example reported of a quaternary carbon center synthesis (Scheme 12). The observations that no reaction was detected in the absence of AgOCl and that only an aza-Michael adduct was observed in the absence of the gold(I) complex led the authors to propose that the silver(I) salt promoted the aza*-*Michael addition and that only the cationic gold(I) complex was associated with the intramolecular hydroalkylation reaction. These observations justified the use of a three-fold excess of AgOCl in relation to the gold complex since the silver salt participated both as a source of a non-coordinating anion OCl^−^ and as an activator of the gold(I) complex in these reactions.

#### Other metals

**Scheme 13:**
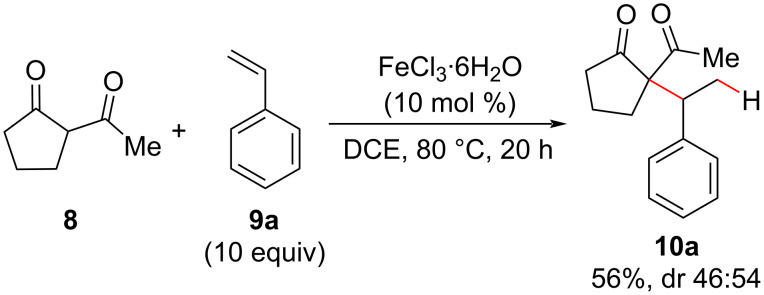
A selected example of quaternary carbon center synthesis using an Fe(III) salt as a catalyst for the intermolecular olefin hydroalkylation (Beller (2007) [46]).

Although gold catalysis predominates in these types of reactions, inexpensive iron salts have also been employed successfully in hydroalkylation reactions. In 2007, Beller and co-workers reported the hydroalkylation of styrenes **9** using FeCl_3_·H_2_O as a source of iron(III) (Scheme 13) [46]. This reaction required higher temperatures than those used in the gold(III) methodology reported previously by Li [38] and a considerable excess (10 equiv) of alkenes (Scheme 6) to achieve the products in practice yields. Despite these disadvantages, this Fe(III) hydroalkylation could be carried out in air instead of an inert atmosphere usually required in gold methodologies.

**Scheme 14:**
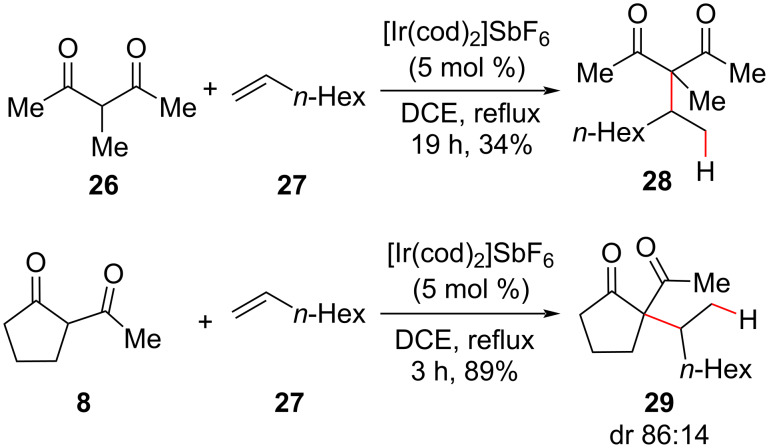
Intermolecular hydroalkylation catalyzed by a cationic iridium complex (Fuji (2019) [47]).

More recently, iridium complexes were used as efficient catalysts in intermolecular hydroalkylation reactions between β-diketones and less reactive alkyl-substituted olefins instead of the aromatic styrenes used in the majority of methodologies (Scheme 14) [47]. Two examples of quaternary carbon centers were described, and a reactivity difference was noted between acyclic **26** and cyclic nucleophile **8** toward the same electrophile. The authors suggested that iridium serves as a π-Lewis acid (although one could propose a C–H activation pathway) that activates the olefin by coordination due to the correlation observed between the nature of the Ir counterion and the reaction yields.

### Radical-based approaches

#### Olefin hydroalkylation via metal hydride atom transfer

**Scheme 15:**
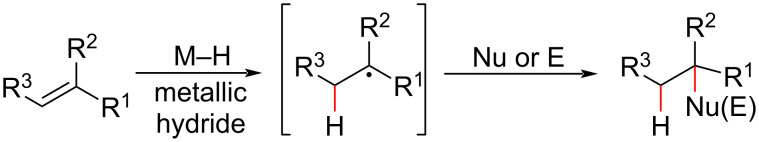
Generic example of an olefin hydrofunctionalization via MHAT (Shenvi (2016) [51]).

Since the mid-2000s and mainly in the present decade, olefin hydrofunctionalization via metal hydride hydrogen atom transfer (MHAT) has gained increasing attention as a powerful tool for the functionalization of non-activated alkenes [48–54]. MHAT involves the transfer of a hydrogen atom from a metallic hydride to an olefin carbon-centered radical that can subsequently be intercepted by a nucleophile or electrophilic species (Scheme 15). One of the synthetic potentials of this methodology lies in the modulation of radical reactivity according to the olefin substituents. Electron-withdrawing groups render the generated radical more susceptible to reaction with electron-rich compounds, whereas substituents that donate electronic density increase the addition reaction rate to electron-deficient ones (Michael acceptors, for example) [55]. Another beneficial aspect of MHAT reactions is the high tolerance to the presence of other functional groups in the reactants due to the mild conditions usually employed.

**Scheme 16:**
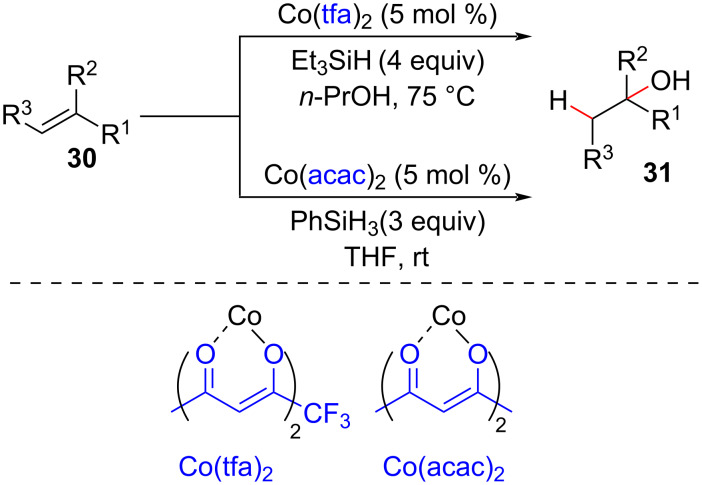
The first examples of olefin hydrofunctionalization run under neutral conditions (Mukaiyama (1989) [56]).

At the end of the 1980s and the middle of the 1990s, Mukaiyama published a series of seminal works reporting the use of silanes as reducing agents (hydride donors) for the hydration of olefins in the presence of cobalt complexes (Scheme 16) [56–58]. These findings enabled the application of neutral conditions, instead of the basic ones that employed borohydrides [59–61], to generate the metal hydride species, thereby expanding the functional group tolerance of these reactions.

In the last 20 years, MHAT methodologies for olefin functionalization have once again spread in the synthetic organic chemistry community, primarily influenced by the work of Carreira [62–66], who employed cobalt complexes in olefin hydro-*N*-functionalization reactions, and by Boger [67–69] on the use of other radical traps in MHAT olefin functionalization reactions. We will now show the evolution of these methodologies, focusing on the construction of all-carbon quaternary centers via olefin hydroalkylation.

#### Olefin hydroalkylations by dimerizations and cycloisomerizations initiated by the MHAT process in all-carbon quaternary synthesis

The synthetic potential of carbon-centered radicals to construct new C(sp^3^)–C(sp^3^) bonds was considerably expanded by the development of MHAT strategies involving olefins as substrates. The regioselective transfer of the hydrogen atom to olefins produces a higher substituted carbon radical (Markovnikov addition), making MHAT methodologies especially useful for quaternary carbon center synthesis.

**Scheme 17:**
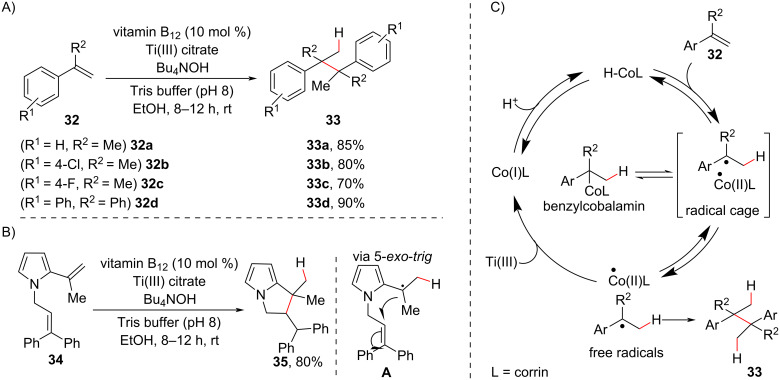
A) Aryl olefin dimerization catalyzed by vitamin B_12_ and triggered by HAT. B) Control experiment to confirm the radical pathway (bottom left). C) Proposed mechanism pathway (Van der Donk (2002) [71]).

One of the first examples of MHAT use in quaternary carbon center construction was the use of vitamin B_12_ (cobalamin) [70] as a catalyst in the reductive dimerization of aryl olefins **32** reported by van der Donk in 2002 [71]. This group showed the formation of a new C–C bond between benzylic carbons, affording compounds **33** with two vicinal quaternary carbon centers (Scheme 17). The occurrence of a radical pathway was proposed based on a control experiment with substrate **34** that had a tethered internal olefin and furnished the expected cyclization product **35**. This reaction indicated a radical cascade initiated by a hydrogen atom transfer from a cobalt hydride to the terminal olefin of **34**, followed by a 5-*exo*-*trig* cyclization (Scheme 17B). The proposed mechanism involves the formation of a cobalt hydride species that, upon transfer of a hydrogen atom to **32**, generates a geminate radical pair containing a Co(II) species and a benzylic radical. The escape from the radical cage then generates the corresponding free radicals, which tend to recombine and eventually generate benzylcobalamin in a parasite pathway. However, the presence of Ti(III) citrate reduces the Co(II) radical, thereby overcoming the “persistent radical effect” observed in cobalt-mediated radical reactions and allowing benzylic radical dimerization to afford **33**.

**Scheme 18:**
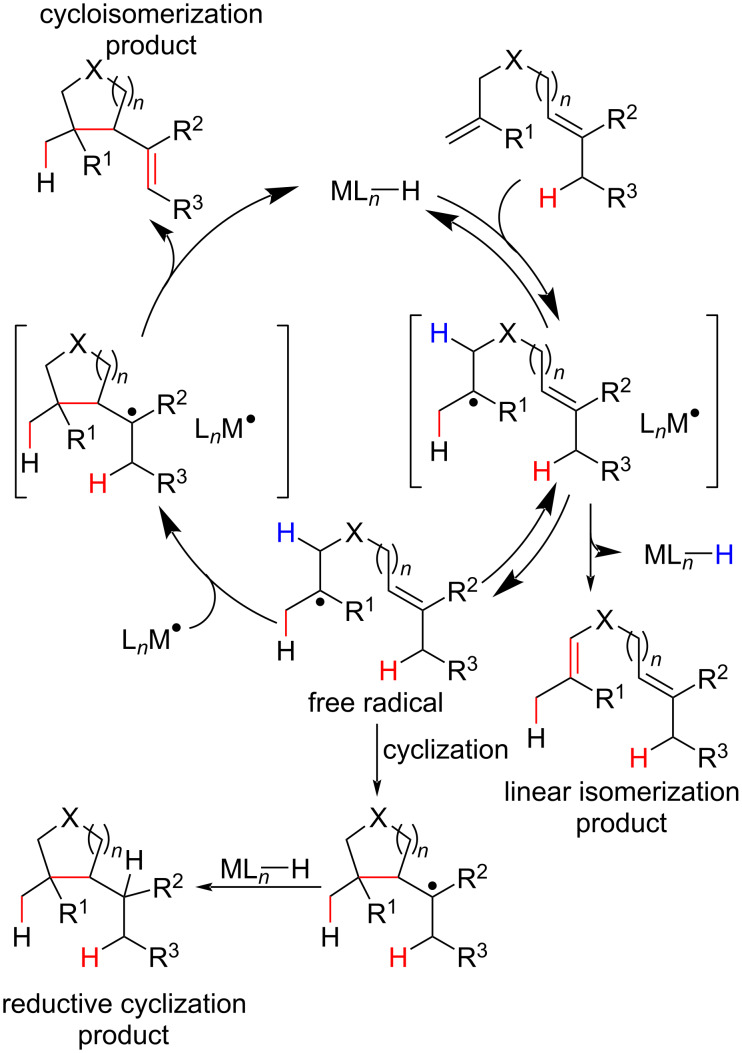
Generic example of MHAT diolefin cycloisomerization and possible competitive pathways. Shenvi (2014) [72].

The efficiency of the solvent radical cage in maintaining the geminate radical pair could be modulated by stereoelectronic changes in the ligands of the transition-metal complexes to favor a desired pathway. One reaction particularity that was sensible to the solvent cage efficiency was the cycloisomerization of diolefins triggered by the MHAT process. Some challenges associated with the development of these reactions were the reversible nature of the HAT and the competition with linear isomerization and reductive pathways (Scheme 18) [72–73].

**Scheme 19:**
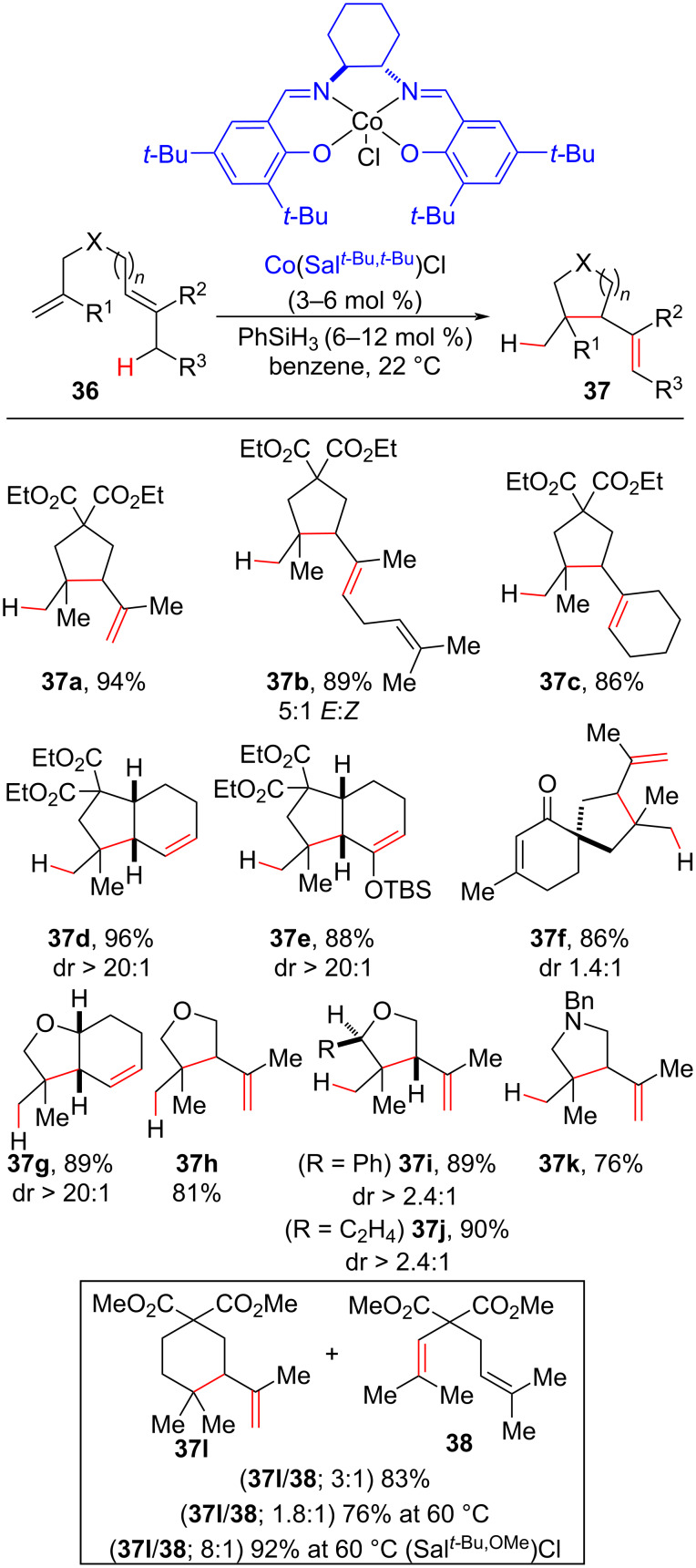
Selected examples of the MHAT-promoted cycloisomerization reaction of unactivated olefins leading to quaternary carbon centers (Shenvi (2014) [72]).

In 2014, the Shenvi group developed an olefin isomerization protocol triggered by an MHAT process using salen cobalt complexes as catalysts to furnish isomerized and cycloisomerized products (Scheme 19) [72]. Five and six-membered carbocycles containing quaternary carbon centers were synthetized in good to excellent yields and with excellent diastereoselectivity in the synthesis of bicyclic compounds (dr > 20:1, Scheme 19, **37d**, **37e**, and **37g**). Substrates containing Lewis base moieties (Scheme 19, **36k**) were tolerated in the reported reaction conditions, thereby representing a synthetic gain over other olefin isomerization methodologies. An important feature of this kind of reaction is the catalytic use of the hydride donor, in this case phenylsilane (PhSiH_3_), due to the regeneration of the active metal hydride species in the cycloisomerization mechanism pathway (Scheme 18).

The lower cyclization constant for the six-membered ring formation, in comparison with the five-membered ones, led to the observation of a mixture of linear and cycloisomerized products when diolefin **36l** was subjected to the optimized conditions. The selectivity was improved when a more electron-rich Salen ligand was employed, highlighting the importance of the ligand structure on the solvent radical cage efficiency and, consequently, on the concentration and “life-time” of the alkyl free radicals generated after the solvent cage collapse. In the latter case, the alkyl radical needed more time to cyclize before engaging in another solvent cage. After a β–H abstraction, it then furnished the cycloisomerized product (Scheme 18).

#### Carbon-centered radical additions via metal hydride hydrogen H atom transfer

Tertiary carbon free radicals are useful intermediates in the construction of new all-carbon quaternary centers. The late stage transition state associated with the formation of C–C bonds involving free radical species means that the new bond is formed at a reasonable distance from the carbon radical acceptor, thereby reducing the expected steric hindrance [74–75]. As mentioned above, a useful strategy for generating the desired tertiary carbon radicals is the use of an MHAT process.

**Scheme 20:**
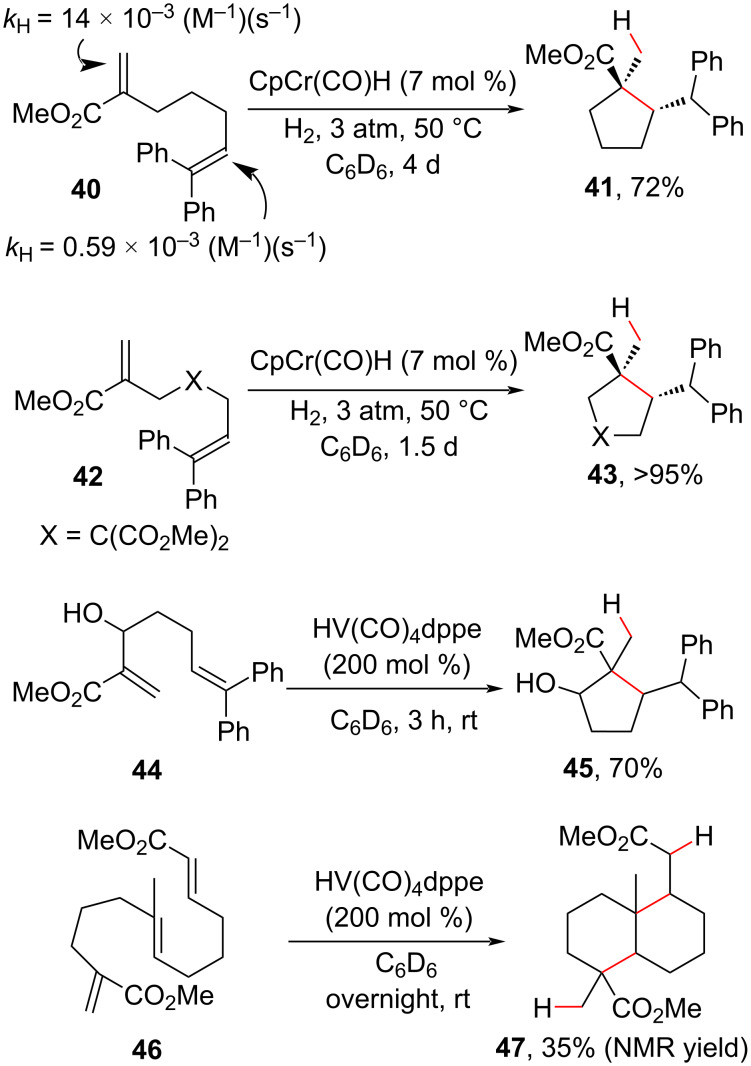
Regioselective carbocyclizations promoted by an MHAT process (Norton (2008) [76]).

In 2008, Norton and co-workers [76] developed a pioneering radical cascade approach based on the generation of carbon free radicals from an MHAT process. The rates (*k*_H_) of hydrogen atom transfer (HAT) from the chromium metal hydride CpCr(CO)_3_H to olefins with diverse substitution patterns and electronic features were measured previously by the rate of deuterium/hydrogen exchange [77], and the observed constants served as a guide to the regioselectivity prediction of the HAT processes in substrates with different olefins. In substrate **40**, for example, the hydrogen atom was transferred preferentially to the terminal carbon of an electron-deficient olefin, yielding **41** in 72% yield after 4 days (Scheme 20). The byproducts of isomerization and reduction were also observed under these conditions, and the authors used the Thorpe–Ingold effect to favor the cyclization product by employing the gem-disubstituted substrate **42** to obtain **43** in excellent yield and with a slightly better reaction time (1.5 days). Under improved conditions using stoichiometric amounts of vanadium hydride, the unprotected Morita–Baylis–Hillman alcohol **44** was subjected to cyclization to furnish **45** in good yield, and a nature-inspired bicycle **47** was synthetized via two sequential 6-*exo*-*trig* cyclizations, giving a preview of the potential use of MHAT methodologies in the synthesis of complex natural products via radical polyene cyclization [78].

Although relevant, the MHAT hydroalkylation methodology developed by Norton employed somewhat unattractive conditions, such as the necessity of maintaining a pressured hydrogen atmosphere in the case of the generation of chromium hydride, the requirement of stoichiometric amounts of vanadium hydride, and the necessity of synthesis and storage (under inert atmospheres) of these metal hydride species. The development of more practical applications of these metal hydride complexes was also discouraged by the long reaction times required.

**Scheme 21:**
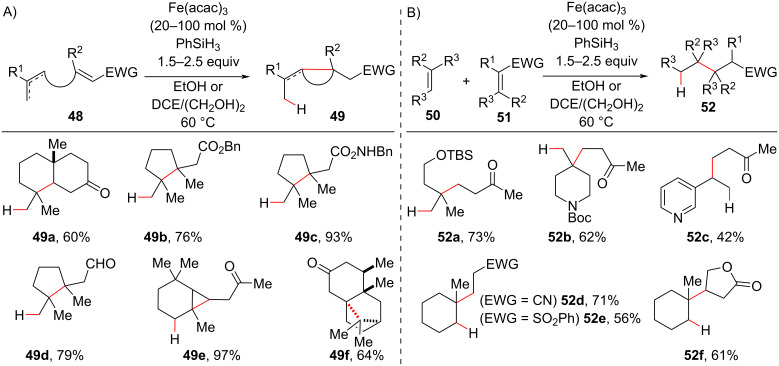
Selected examples of quaternary carbon centers synthetized via intra- (A) and intermolecular (B) MHAT hydroalkylation reactions under mild conditions (Baran (2014) [79]).

In 2014, Baran developed a hydroalkylation methodology that occurred under much milder conditions. For example, the reactions could be conducted in an air atmosphere, and the reaction times were shorter using a combination of Fe(acac)_3_ and PhSiH_3_ as the reductor (Mukaiyama-like conditions) in the presence of an alkoxide source (Scheme 21) [79]. Under these conditions, the radical formed after the HAT with unactivated olefins reacted with electron-deficient alkenes (Giese-type addition) to form new C–C bonds in intra- and intermolecular fashions. The authors reported a great diversity of electron-deficient alkenes, including α,β-unsaturated carbonyl compounds like the usually less reactive acrylamide derivative **48c** acrylonitrile. Six- (**49a**), five- (**49b–49d**), and three-membered (**49e**) cyclization reactions were carried out under these conditions to afford compounds with quaternary centers. Notably, the highly sterically congested quaternary center **49f** could be synthesized in an intramolecular reaction. The reactions were also tolerable to functional groups, such as silyl ethers (**52a**), carbamates (**52b**), and heterocycles (**52c**), present in the olefin moiety.

**Scheme 22:**
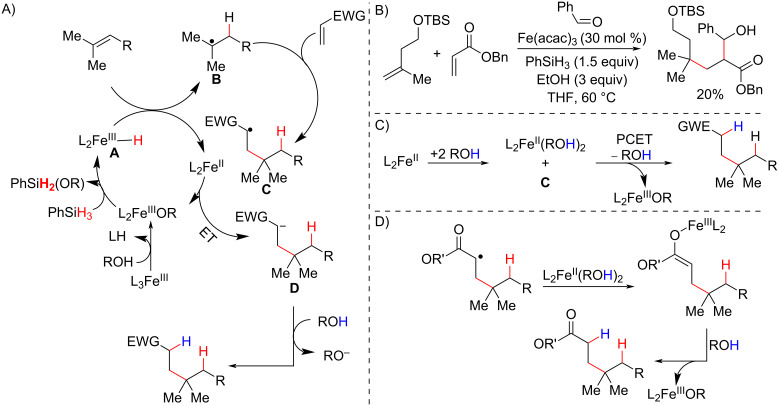
A) Proposed mechanism for the Fe(III)/PhSiH_3_-promoted radical conjugate addition between olefins and electron-deficient olefins via a MHAT process (Baran (2014, 2017) [79–80]). B) Control experiment to support the ET hypothesis (Baran (2017) [80]). C) Proton-coupled electron transfer (PCET) pathway and D) *O*-bounded Fe(III) enolate formation as alternative pathways to explain the proton transfer step (Holland (2019) [81]).

The initial mechanism proposed by the authors begins with the formation of Fe(III) hydride species (**A**), followed by hydrogen atom transfer to the olefin in a Markovnikov-type addition to generate a carbon-centered radical (**B**) that attacks the electron-deficient olefin (Scheme 22A). The newly formed alkyl radical (**C**) is then reduced by a Fe(II) species to an enolate (**D**) in an electron transfer (ET) step; a proton abstraction then delivers the hydroalkylated product. A very clever experiment was designed by Baran and Holland [80] to corroborate the hypothesis of enolate **D** formation by the Fe(II) reduction of alkyl radical **C**. They performed an intermolecular hydroalkylation in the presence of benzaldehyde, and then trapped the putative enolate intermediate in an aldol reaction (Scheme 22B), which was confirmed by the observation of the aldol product in low yield.

The ET step initially proposed by Baran has undergone insightful discussions about its nature, and a recent theoretical study postulated a proton-coupled electron transfer (PCET) pathway (Scheme 22C), in which an alcohol bound to an Fe(II) species transfers the proton to the alkyl radical in a concerted way, or the combination of the radical **C** with another Fe(II) species to generate an *O*-bounded Fe(III) enolate that then abstracts a proton of the alcohol (Scheme 22D). These pathways were deemed more probable than the ET proposal, especially for radicals derived from alkyl radical additions to acrylates due to their lower oxidation potentials toward the Fe(II) species [81].

**Scheme 23:**
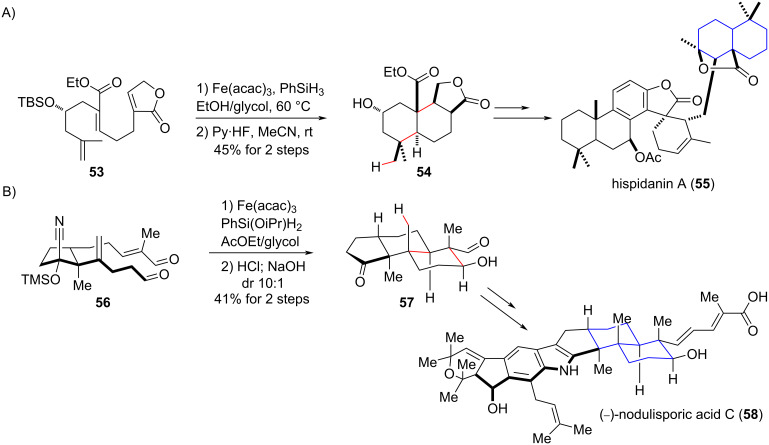
Examples of cascade reactions triggered by HAT for the construction of *trans*-decalin backbone unities (highlighted in blue) in the total synthesis of natural products. A) Radical conjugate addition/Michael addition cascade (Liu (2017) [83]). B) Radical conjugate addition/aldolization cascade (Pronin (2018) [84]).

The methodology developed by Baran had a rapid impact on the organic synthesis community, and numerous examples of this strategy in total synthesis can be found [82–90]. The reaction’s mild conditions and high chemoselectivity allowed its use even in advanced steps in a total synthesis route when diverse functional groups were present in the intermediates. Two representative examples of the potential of this strategy in total synthesis routes are the work by Liu [83] for the synthesis of hispidanin A (**55**, Scheme 23A) and the synthesis of (−)-nodulisporic acid C (**58**) described by Pronin [84] (Scheme 23B). In both studies, cascade reactions involving the putative enolate ion formed after the radical conjugate addition triggered by the HAT process (see Scheme 22 intermediate **D**) allowed the construction of complex polycyclic units containing quaternary carbon centers. In the first case, a cascade radical addition/conjugate addition promoted by a HAT process to the terminal olefin of **53** (Scheme 23A) afforded *trans*-decalin **54** as the major diastereomer [83]. In the second case, a radical addition/aldolization was successfully developed to afford another *trans*-decalin unity as a major diastereomer in a 10:1 diasteroisomeric ratio (Scheme 23B, **57**) [84]. Notably, in both cases, four contiguous stereogenic centers were constructed by this cascade reaction.

**Scheme 24:**
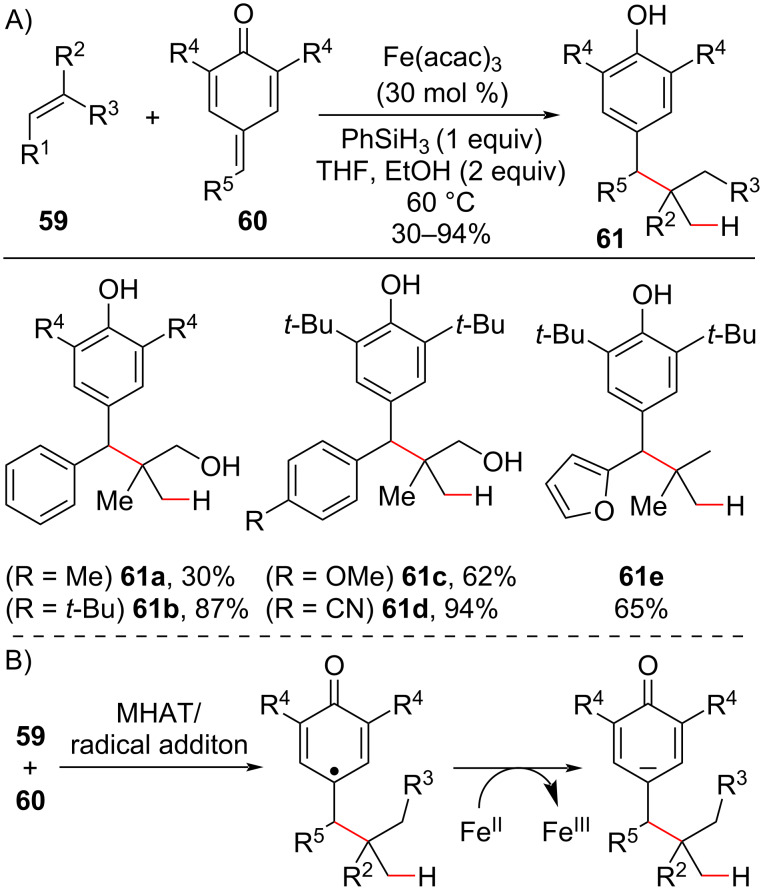
A) Selected examples of the MHAT-promoted radical conjugate addition between olefins and *p*-quinone methides to afford alkylated phenolic compounds containing quaternary carbon centers. B) Mechanism steps to generate the enolate intermediate (Cui (2016) [91]).

As exemplified above (Scheme 23), the hypothesized enolate intermediate produced in the radical conjugate addition promoted by a MHAT process could be engaged in sequential reactions, offering a range of possibilities for the design of new hydroalkylation cascades. In 2016, the Cui group reported the hydroalkylation of olefins **59** using *p*-quinone methides **60** as electrophilic partners (Scheme 24A) [91]. Alkenyl alcohols could be employed without needing to protect the hydroxy functional group, and the acidity of the phenolic hydrogen present in the product was also compatible with the reaction conditions. Alkylated phenols **61** were obtained after protonation/isomerization of the generated enolate intermediate (Scheme 24B).

**Scheme 25:**
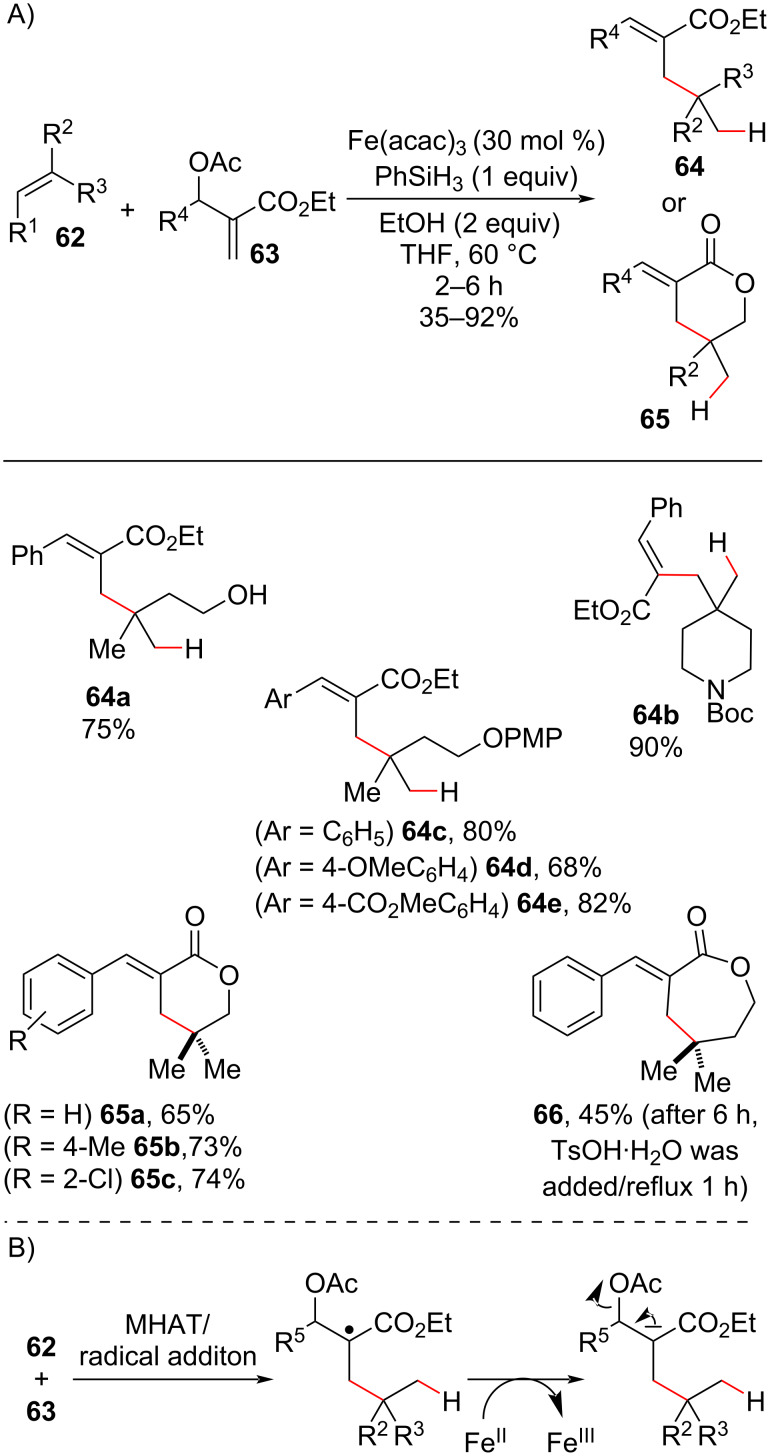
A) MHAT triggered radical conjugate addition/E1cB/lactonization (in some cases) cascade between olefin and MBH acetates to afford hydroallylation products containing quaternary carbon centers. B) Simplified mechanism. Cui (2016) [92].

In 2018, the same authors continued to explore the synthetic opportunities offered by the enolate generated in MHAT radical additions [92]. Using MBH acetates **63** as substrates, olefin hydroallylation products **64** were obtained via E1cB elimination of the acetate leaving group driven by the enolate intermediate (Scheme 25B). Six-membered lactones (Scheme 25A, **65a–c**) were obtained in a cascade reaction when alkenyl alcohols were employed, and an example of a seven-membered lactone was reported (Scheme 25A, **66**). However, in the latter case, acid catalysis and reflux were needed to ensure cyclization.

**Scheme 26:**
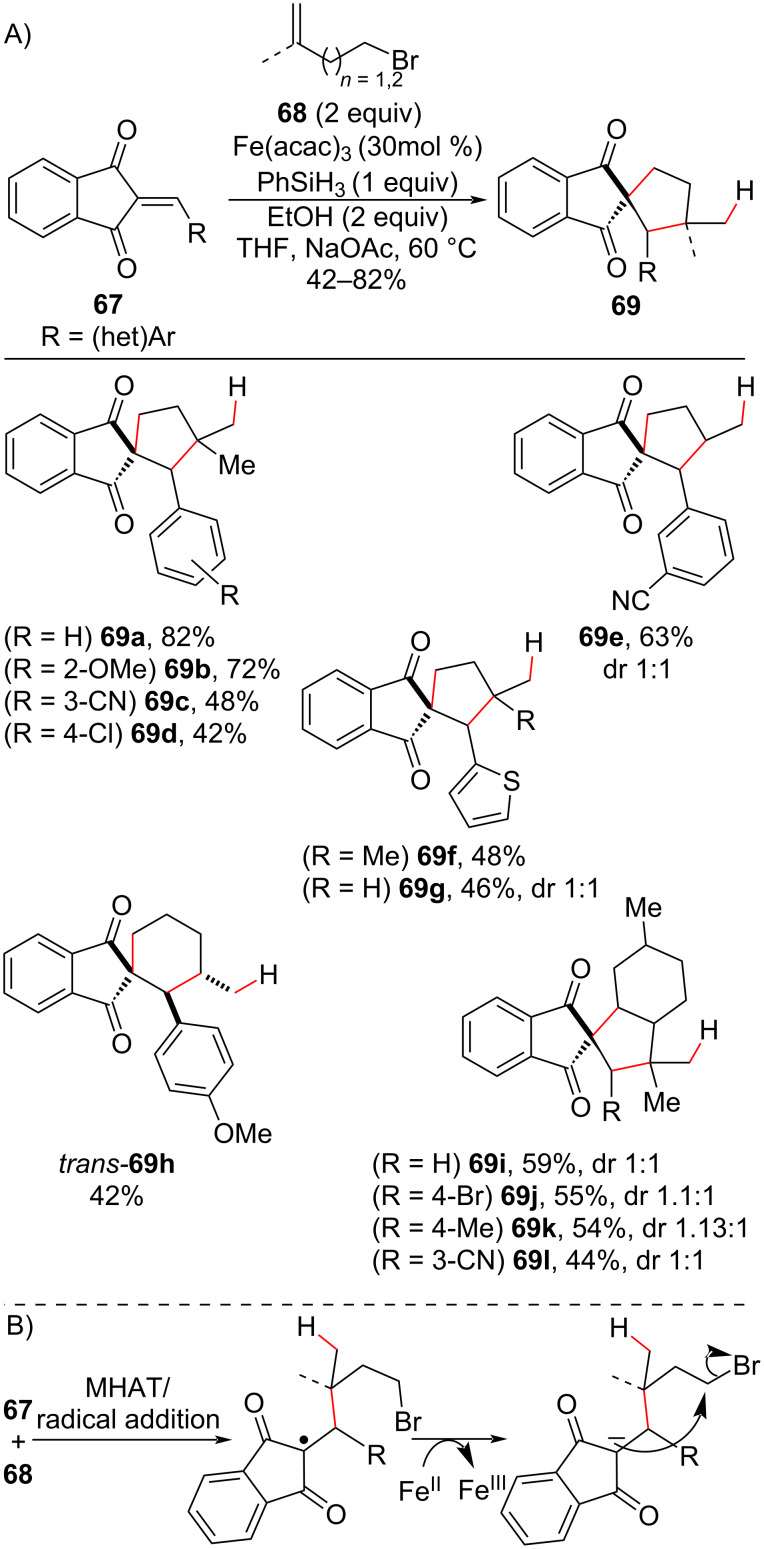
A) Spirocyclization promoted by Fe(III) hydroalkylation of unactivated olefins. B) Simplified mechanism (Cui (2018) [93]).

Still taking advantage of the enolate intermediate, the Cui group reported the use of olefins **68**, containing tethered leaving groups in Fe(III)-promoted hydroalkylation/cyclization cascades (Scheme 26A) [93]. Here, the carbocyclization occurred via a S_N_2 mechanism between the enolate intermediate and the tethered halide (Scheme 26B). Using arylidene diones **67** as radical acceptors, spiro compounds **69** were obtained in moderate to good yields. Five- and six-membered rings were synthesized by this method, and the tetracyclic compounds **69i–l** were obtained when isopregol halide derivatives were employed. No diastereoselectivity was observed in the five-membered cyclizations (Scheme 26A, **69e–g**), whereas the six-membered ring **69h** was obtained exclusively as the *trans* diastereoisomer (Scheme 26B).

**Scheme 27:**
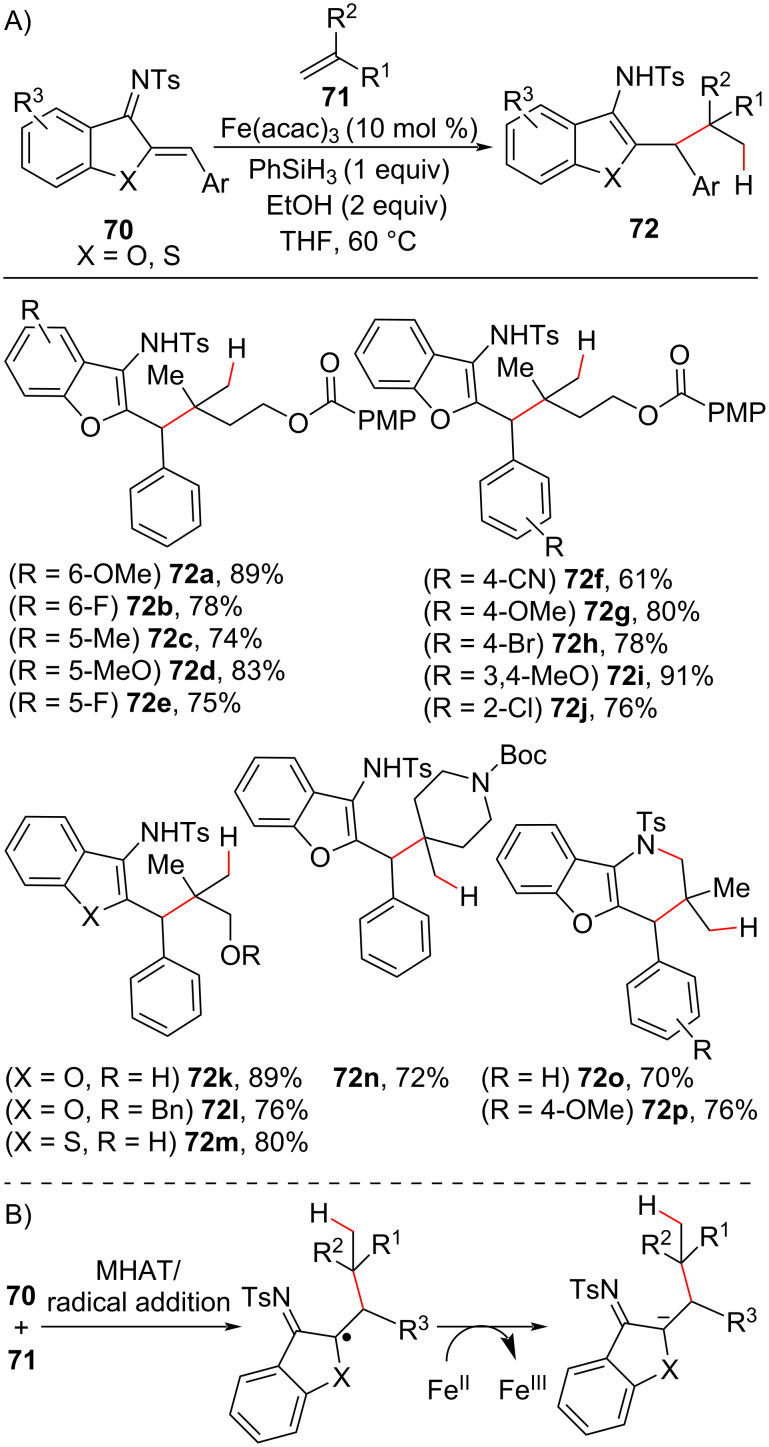
A) Selected examples of the construction of a carbon quaternary center by the MHAT-triggered radical addition between olefins and unsaturated imines. B) Mechanism for the formation of the key aza-enolate intermediate (Xu (2019) [94]).

Based on the previous work using the Fe(III)/silane system to promote hydrogen atom transfer to olefins, the Xu group explored the use of unsaturated imines **70** as radical acceptors in MHAT-driven radical conjugate additions with olefins [94]. This approach allowed the synthesis of alkylated benzofurans and benzothiophenes (Scheme 27). Investigations of the substrate scope revealed that both electron-donating and electron-withdrawing group substituents at the aromatic portions of the imine substrate could afford the aromatic heterocycle in good yields (Scheme 27A, **72a–j**). However, better yields were observed with substrates with aromatic electron-donor substituents (**72a** and **72i**). Other olefin-tethered functional groups were tolerated under the reaction conditions (Scheme 27A, **72k–n**), and *S*-containing imines also delivered the alkylated benzothiophenes in good yield (**72m**). The use of olefins bearing leaving groups such as halides also afforded cyclic products (Scheme 27; **72o** and **72p**). Mechanistically, the alkylated heteroaromatic compounds were obtained after protonation/isomerization of the aza-enolate intermediate formed in this type of reaction (Scheme 27B).

**Scheme 28:**
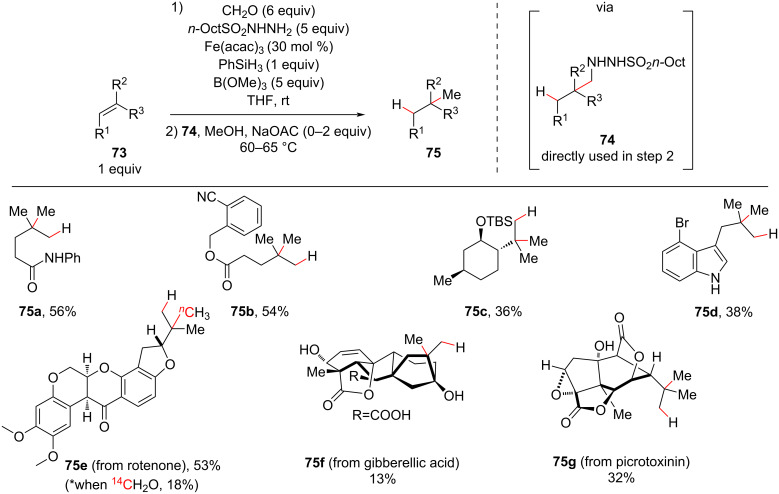
Hydromethylation of unactivated olefins under iron-mediated MHAT (Baran (2015) [95]).

The nucleophilic alkyl radical intermediate generated under MHAT conditions offers an array of trapping possibilities for the formation of new carbon–carbon bonds. Baran and co-workers further expanded the potential of olefin functionalization under MHAT conditions by trapping the alkyl radical with formaldehyde hydrazone, which resulted in hydrazide **74** that could afford formal hydromethylated compounds **75** after the extrusion of nitrogen and sulfinic acid (Scheme 28) [95]. The hydrazone was generated in situ due its instability, and a simple exchange of the solvent from THF to methanol and gentle heating was necessary to optimize the formation of **75** from **74**. As is usual in these Mukaiyama-like reaction conditions, the developed hydromethylation tolerated an array of functional groups and the late-stage functionalization of complex natural products (**75e–g**). Deuterated and other isotopically-labeled hydrazides could also be employed. Notably, this methodology considerably shortened the existing synthetic routes for adding a methyl group to olefins.

**Scheme 29:**
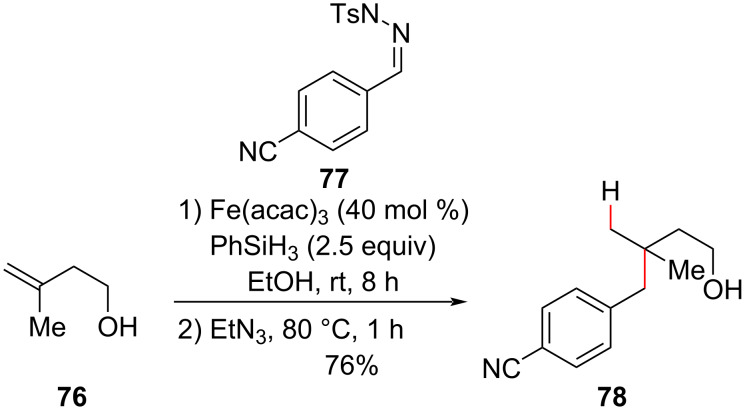
The hydroalkylation of unactivated olefins via iron-mediated reductive coupling with hydrazones (Bradshaw (2020) [98]).

More recently, the Bradshaw group has been exploring the potential use of tosyl hydrazones in reductive couplings with olefins initiated via metal hydride atom transfer [96–97]. In 2020, they realized that formal hydroalkylated products could be obtained by coupling substituted tosyl hydrazones with olefins (Scheme 29) [98], similarly to the hydromethylation protocol developed by Baran [95] (Scheme 28). The higher stability of the substituted hydrazones obviated their generation in situ*,* and as ethanol was identified as a better solvent in the initial coupling step, no solvent exchange was necessary in the fragmentation step of the hydrazide intermediate. The addition of triethylamine and heating the reaction to 80 °C for 1 hour were sufficient. The reaction tolerated (hetero)aryl and aliphatic tosyl hydrazones as radical acceptors and a variety of olefins with different electronic natures as nucleophilic partners (i.e.*,* vinyl ethers, sulfides, and acetamides). One example of the synthesis of a quaternary center was reported by the authors (Scheme 29).

**Scheme 30:**
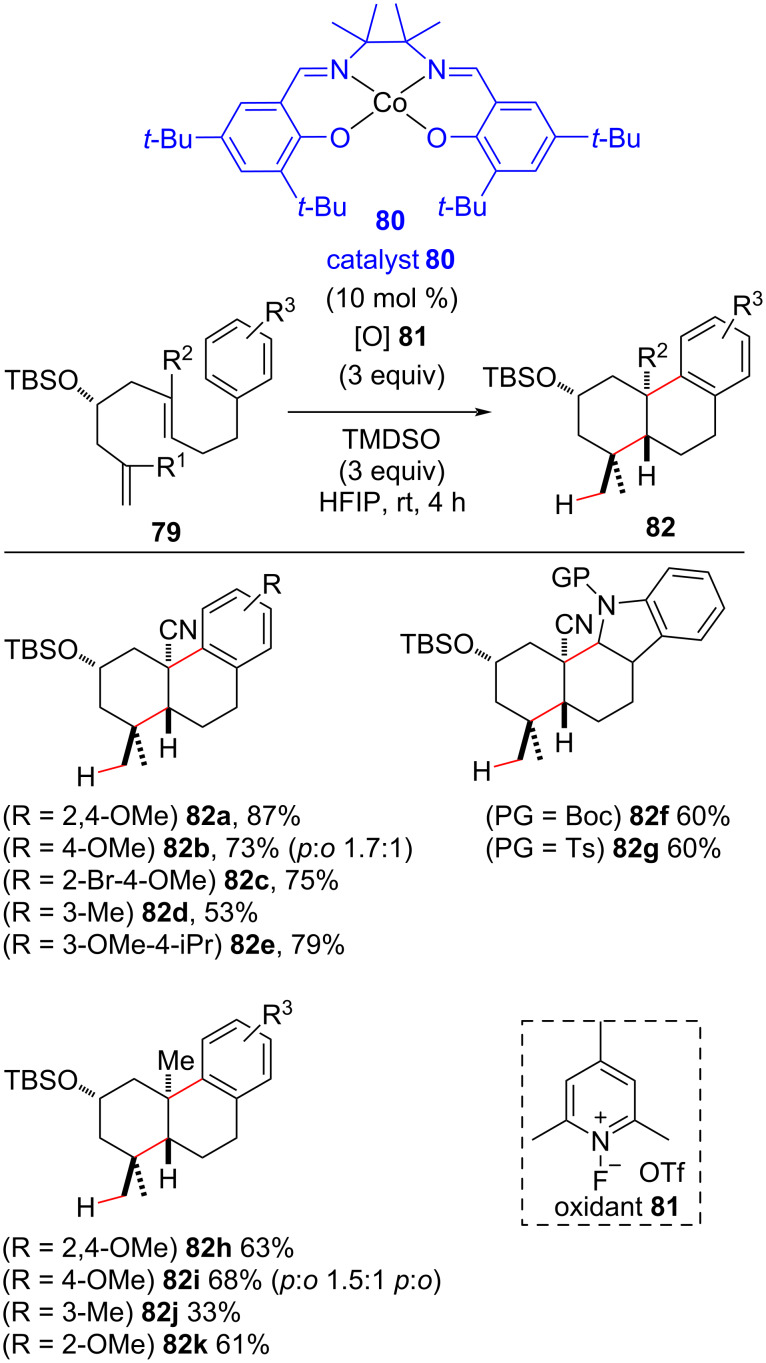
Selected examples of the Co(II)-catalyzed bicyclization of dialkenylarenes through the olefin hydroalkylation/arylation cascade (Vanderwal (2020) [99]).

**Scheme 31:**
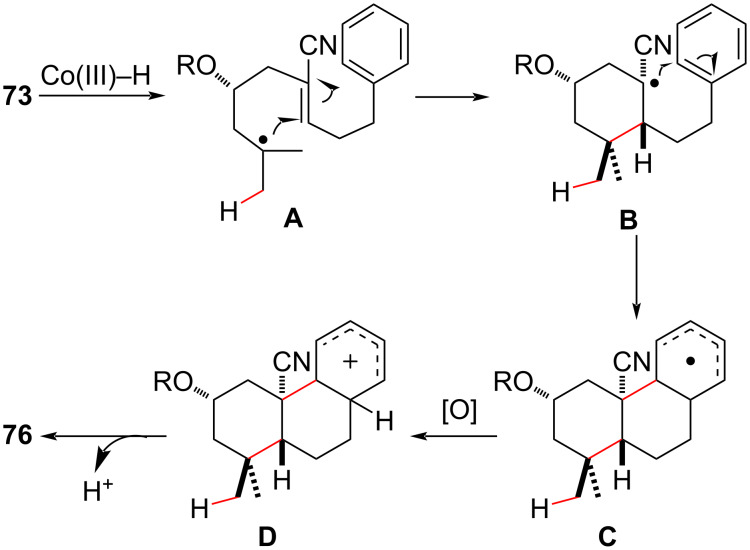
Proposed mechanism for the bicyclization of dialkenylarenes triggered by a MHAT process (Vanderwal (2020) [99]).

The latter steps of reductive coupling cascades of unactivated olefins with electron-deficient olefins under MHAT conditions involve the reduction of the radical generated at the α-position of an EWG to an enolate that could be engaged in further reactions (i.e., Scheme 23 to Scheme 27). Recently, Vanderwal developed an oxidative cobalt-catalyzed olefin hydroalkylation/arylation cascade in which, instead of an EWG olefin as a terminator of the radical cascade, an aryl moiety served to this goal to generate a stabilized aryl radical that was further oxidized by an external oxidant (Scheme 30) [99]. Bicycle units **82** founded in natural products could be synthetized under Co(II) salen complex **80** catalysis at room temperature with excellent stereoselectivity. Only *trans*-decalins **82** were observed in the presence of secondary OTBS groups in the substrate backbone; this was not observed in the Fe(III)-polyene cyclization cascades reported by Liu [83] and Pronin [84] (see Scheme 23). Notably, two quaternary carbon centers were constructed sequentially, and substrates containing electron-rich aromatic moieties afforded bicyclization products in better yields than were obtained with those with less activated phenyl unities. However, electron-withdrawing groups were not suitable substrates for this reaction, most probably because of the lower stability of the cyclohexadienyl cation intermediate formed (see Scheme 31, intermediate **D**). A replacement of the phenyl moiety with *N*-protected indoles led to reduced indole products (indolines **82f** and **82g**). The substitution of the acrylonitrile group for an acrylate unit completely inhibited the reaction, whereas the replacement with an unactivated trisubstituted olefin unit led to cyclized products in lower yields (Scheme 30, **82h–k**).

The most likely mechanism starts with a 6-*exo*-*trig* cyclization step via radical conjugate addition triggered by a MHAT process that results in the formation of a stabilized tertiary radical **B** (Scheme 31). The cyclization between the radical **B** and the aromatic ring then leads to the stabilized radical **C**, which undergoes an oxidation process to generate carbocation **D**. A proton abstraction from **D** then affords the observed product.

#### Cross-coupling reaction between unactivated olefins and alkyl halides under nickel catalysis

The use of alkyl halides in transition-metal-catalyzed cross-couplings to form new C(sp^3^)–C(sp^3^) bonds remains a formidable challenge. The main issues associated with obtaining these cross-coupling products are the tendency of the alkylmetal intermediate generated during the reaction to undergo a β-hydride elimination and the slow rate of the oxidation step [100–101]. Nickel catalysis is a viable alternative for this kind of cross-coupling reactions due to its particular radical mechanism, instead of the common metal-oxidative addition to organohalides [102–104]. Examples of the coupling between primary, secondary, and even tertiary alkyl halides with nucleophilic species, such as alkyl Grignard, alkyl zinc, and alkylborane species, can be found in the literature [105–106].

A common drawback of these Ni cross-coupling methodologies is the use of nucleophile partners with a low tolerance to other functional groups present in the substrate, as is the low stability of some of them [105–106]. The combination of Ni complexes with silyl reductants could avoid these issues by transforming the olefins in the nucleophile partners in cross-coupling reactions under mild conditions [107].

**Scheme 32:**
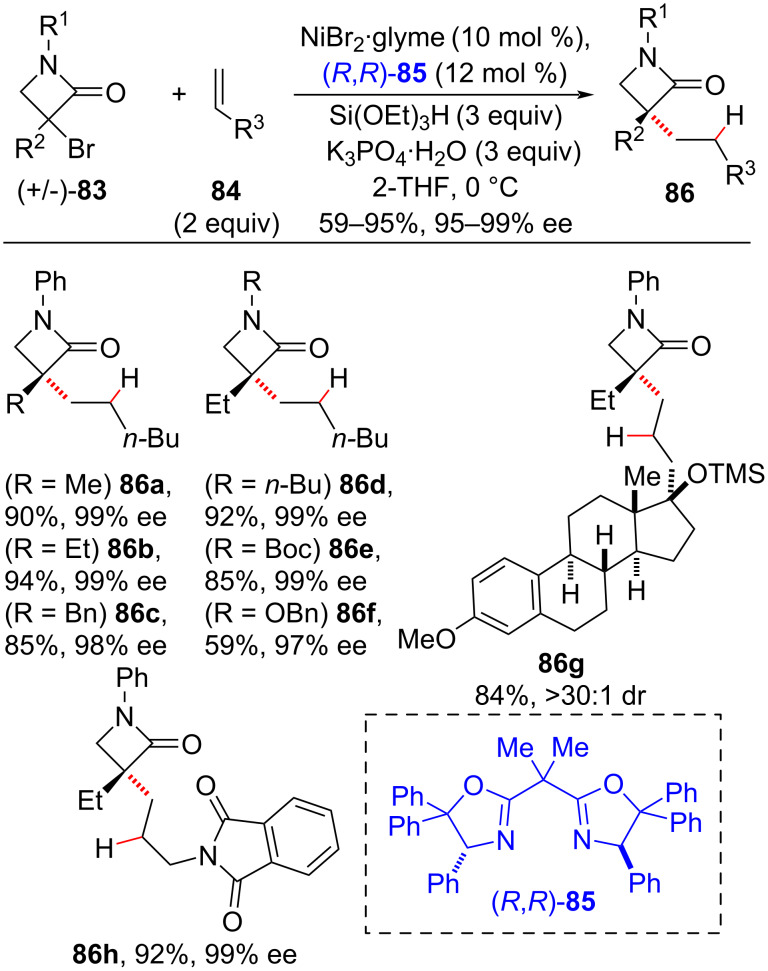
Enantioconvergent cross-coupling between olefins and tertiary halides (Fu (2018) [108]).

In 2018, the Fu group developed an enantioconvergent cross-coupling between olefins **84** as nucleophile partners and racemic secondary and tertiary α-bromo-*N*-protected β-lactams **83** under nickel catalysis, along with the chiral bis(oxazoline) ligand **85** and triethoxysilane (Scheme 32) [108]. Substrate structural variations on **84** had only a small impact on the reaction stereoselectivity, and olefin **84h** containing a biologically relevant steroid moiety was successfully hydroalkylated under these conditions. This confirmed the potential of this strategy to forge challenging carbon sp^3^–sp^3^ bonds.

**Scheme 33:**
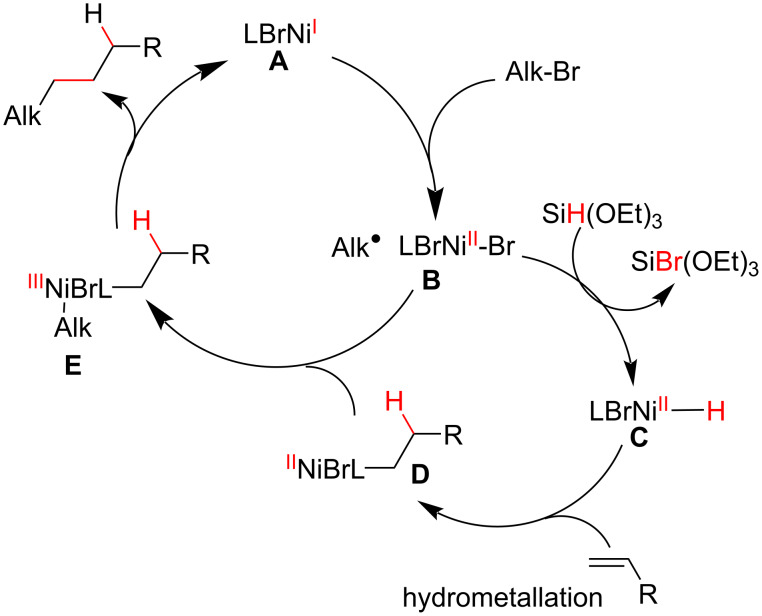
Proposed mechanism for the Ni-catalyzed cross-coupling reaction between olefins and tertiary halides (Fu (2018) [108]).

The proposed mechanism starts with a Ni(I) species (**A**), which acts as a radical initiator toward the alkyl halide to generate an alkyl radical and the Ni(II) complex (**B**) (Scheme 33). The reduction of **B** by the organosilane produces Ni(II) hydride (**C**), which complexes with the olefin to afford, after a β-migratory insertion, the alkyl–Ni complex (**D**) with anti-Markovnikov selectivity. Addition of the alkyl radical formed in the first step adds to **D**, followed by a reductive elimination step then affords the cross-coupling product.

**Scheme 34:**
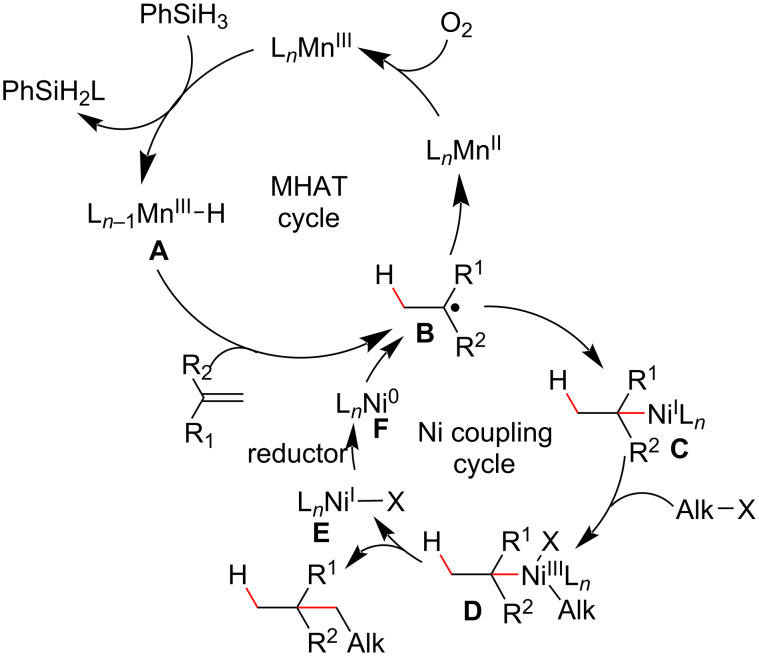
Proposed catalytic cycles for a MHAT/Ni cross-coupling reaction between olefins and halides (Shenvi (2019) [109]).

The enantioconvergent cross-coupling developed by Fu was restricted to the use of “activated” halides, such as the α-halocarbonyl compounds **83** (Scheme 32), and only linear products could be achieved. In 2019, Shenvi proposed a metal cooperative catalysis system to promote a MHAT/Ni cross-coupling between simple halides and unactivated olefins, thereby obtaining branched cross-coupling products containing quaternary carbon centers [109]. The reaction design was based on the interception of tertiary alkyl radicals (**B**) from a hydrogen atom transfer process involving olefins by a low valent Ni complex (**F**), generating an alkyl–Ni complex (**C**) that, upon an oxidative addition to alkyl halides, gives the Ni(III) complex (**D**) (Scheme 34). After a reductive elimination step, a branched cross-coupling product was observed, and the Ni(I) species formed was reduced to generate the catalytically active Ni(0) species (**F**).

**Scheme 35:**
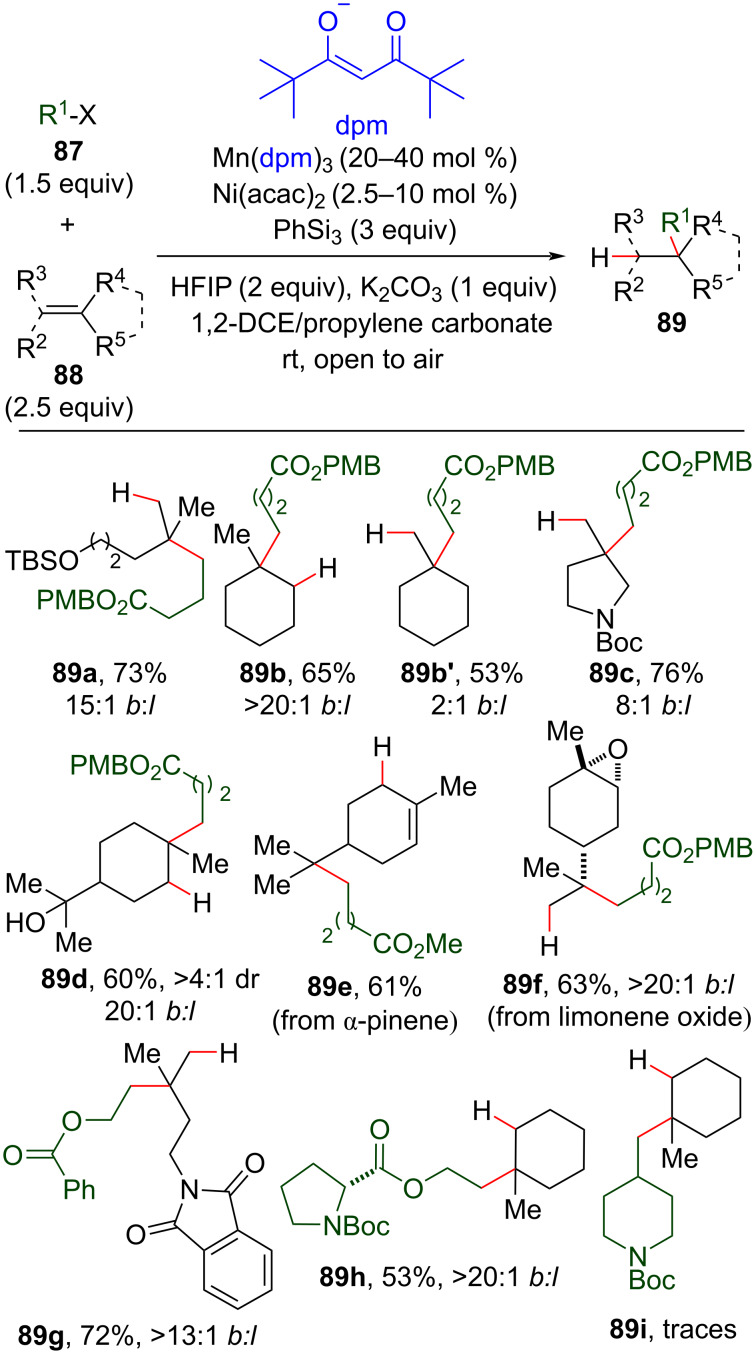
Selected examples of the hydroalkylation of olefins by a dual catalytic Mn/Ni system (Shenvi (2019) [109]).

The same authors then employed a catalytic system composed of Mn(III)tris(dipivaloylmethane) and phenylsilane in the MHAT cycle and Ni(II)bis(acetylacetonate) in the cross-coupling cycle. They achieved the desired cross-coupling products **89** with the reaction conducted in open air and under mild conditions (Scheme 35) [109]. Notably, the use of propylene carbonate as the co-solvent removed the need for inclusion of reductant or oxidant additives, such as Mn(0) and MnO_2_, that had been employed in the previous work by the same authors to ensure catalytic turnover [110]. Substrates containing functional groups, like esters, phthalimides, silyl enol ethers, boronates, ketones, tertiary alcohols, epoxides, and cyclobutanes, were compatible with the reaction conditions. Linear cross-coupling products, such as those described by Fu [108], were also observed, putatively derived from a hydrometallation step mediated by a Ni-hydride species (see intermediate **D**, Scheme 33). The ratio between branched and linear products was strongly associated with the olefin structure. Acyclic and endocyclic trisubstituted olefins, in general, afforded branched cross-coupling products with high selectivity (Scheme 35, compounds **89a**, **89b**, **89d**, **89f–h**), whereas exocyclic trisubstituted olefins led to a lower ratio between the regioisomers (Scheme 35, **89b’** and **89c**). Natural products like α and β-pinene that contain an olefinic moiety were also used as substrates, and these afforded ring-opened products like **89e**, thereby corroborating a radical mechanism hypothesis [111]. Enantiopure substrates, like limonene oxide (**88f**) and the proline derivative **88h**, afforded the hydroalkylated product without erosion of the enantiomeric excess. Congested halides, such as **87i**, were poor substrates for this transformation, and low conversions (1–10%) were observed when more sterically demanding halides were employed.

#### Reductive atom transfer reactions

Reductive atom transfer reactions (reductive ATR), in which carbon-centered radicals are added to the olefin double bond, followed by a hydrogen abstraction, are a complementary approach to the MHAT methodologies and allow the generation of anti-Markovnikov hydroalkylation products [112–116]. The polar complementarity observed in radical addition reactions necessitated that electron-deficient carbon-centered radicals first are generated in these ATR additions to olefins [117]. A common drawback of this kind of reaction is the poor reactivity of non-terminal olefins due to their tendency to form non-productive allylic radicals via hydrogen atom transfer reactions.

**Scheme 36:**
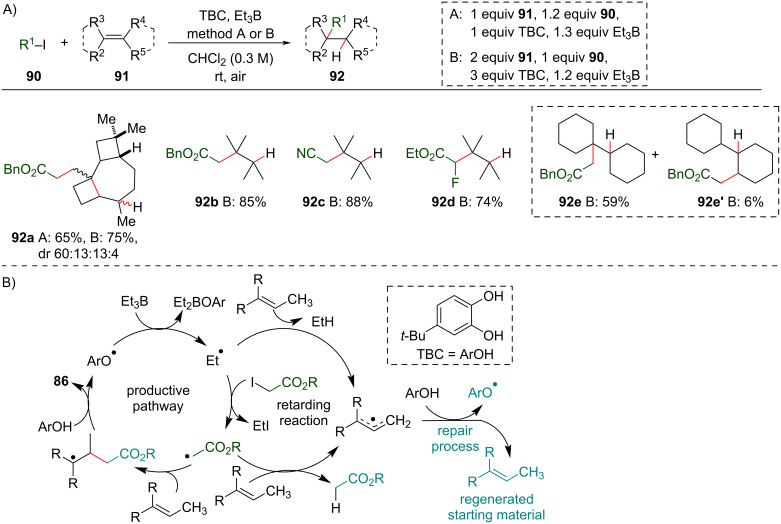
A) Selected examples of quaternary carbon center synthesis by reductive atom transfer; TBC: 4-*tert*-butylcatechol. B) Proposed reaction mechanism showing the radical chain repair performed by TBC (ArOH) (Renaud (2018) [118]).

In 2018, the Renaud group proposed an elegant solution to circumvent this problem by employing 4-*tert*-butylcatechol (TBC) as an external hydrogen atom source to regenerate the starting material under transition-metal-free conditions [118]. Employing α-EWG-substituted iodides as radical precursors and the triethylborane/TBC system as a radical initiator, the authors obtained anti-Markovnikov hydroalkylation products **92** under mild reaction conditions (room temperature and under air atmosphere). Terminal and non-terminal alkenes were successfully functionalized, and different α-EWG-substituted iodides were compatible with the reaction. Among the examples reported, five demonstrated the capacity of the methodology for constructing quaternary carbon centers (Scheme 36A). The hydroalkylation of bicyclohexyl (**91e**) with benzyl iodoacetate revealed another hydroalkylation product **92e’**, indicating a possible double bond isomerization under these reaction conditions.

The postulated mechanism starts with the generation of an ethyl radical from BEt_3_. This radical then acts as a radical initiator to promote the homolytic cleavage of the carbon–halogen bond of the α-EWG-substituted iodides (Scheme 36B). The radical addition of the electron-poor carbon-centered radical formed to the olefin double bond then leads to another radical intermediate that, after hydrogen abstraction from TBC, affords **92**. The ethyl and the α-EWG-substituted radicals formed in the initial steps could engage in a retarding radical pathway with the olefin to generate an allyl radical species via H abstraction. TBC acts on this species through hydrogen atom transfer to regenerate the olefin double bond and allows its reentry into the productive catalytic cycle (a repair chain step), thereby allowing the olefins that are less reactive, such as the non-terminal ones, to be effectively functionalized. Eventually, the repair chain step could lead to an isomerized olefin. However, in most cases, it proceeds with almost perfect regioselectivity toward the regeneration of the starting material.

**Scheme 37:**
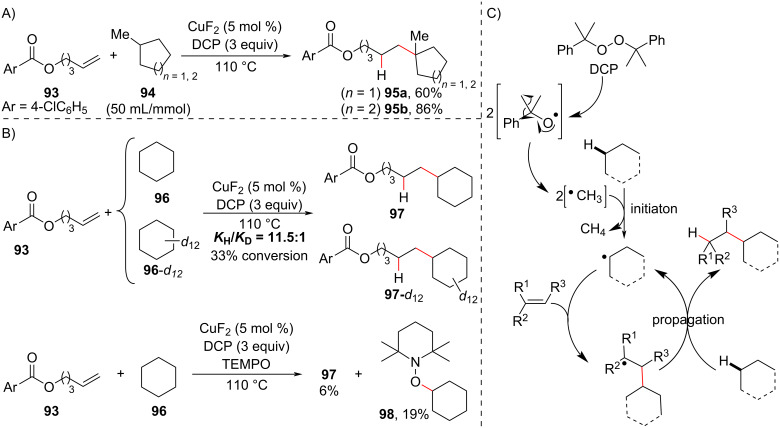
A) Selected examples of quaternary carbon centers synthetized by radical addition to unactivated olefins promoted by selective alkane C(*sp*^3^)–H bond cleavage. B) Control experiments. C) Proposed mechanism (Liu (2018) [119]).

Another example of the potential of ATR in olefin hydrofunctionalization is the methodology developed by the Liu group [119], in which a carbon-centered radical is generated from simple alkanes instead of the more usual halogenated compounds. The authors reported that, in the presence of dicumyl peroxide (DCP) as a radical initiator and a copper salt as an additive, the reaction between unactivated olefins and alkanes (in large excess, 50 mL/mmol) afforded anti-Markovnikov hydroalkylation products at high temperature (110 °C) (Scheme 37). Two examples of the synthesis of quaternary carbon centers were reported using methylcycloalkanes (**94a** and **94b**) and the terminal olefin **93** (Scheme 37A). Both examples showed perfect regioselectivity for the functionalization of the tertiary C(sp^3^)–H bond.

Mechanistic studies carried out by the authors revealed a strong kinetic isotope effect (KIE = 11.5:1) when a competitive reaction was carried out between **96** and completely deuterated cyclohexane **96**-*d*_12_. A considerable decrease in the reaction yield was observed when the reaction was carried out in the presence of radical trapping reagents (Scheme 37B). Based on these observations, the C(sp^3^)–H homolytic bond cleavage appears to be the reaction rate-determining step in a radical mechanism in which the alkane participates in both the initiation and propagation steps of the radical chain (Scheme 37C).

#### Photoinduced electron transfer

**Scheme 38:**
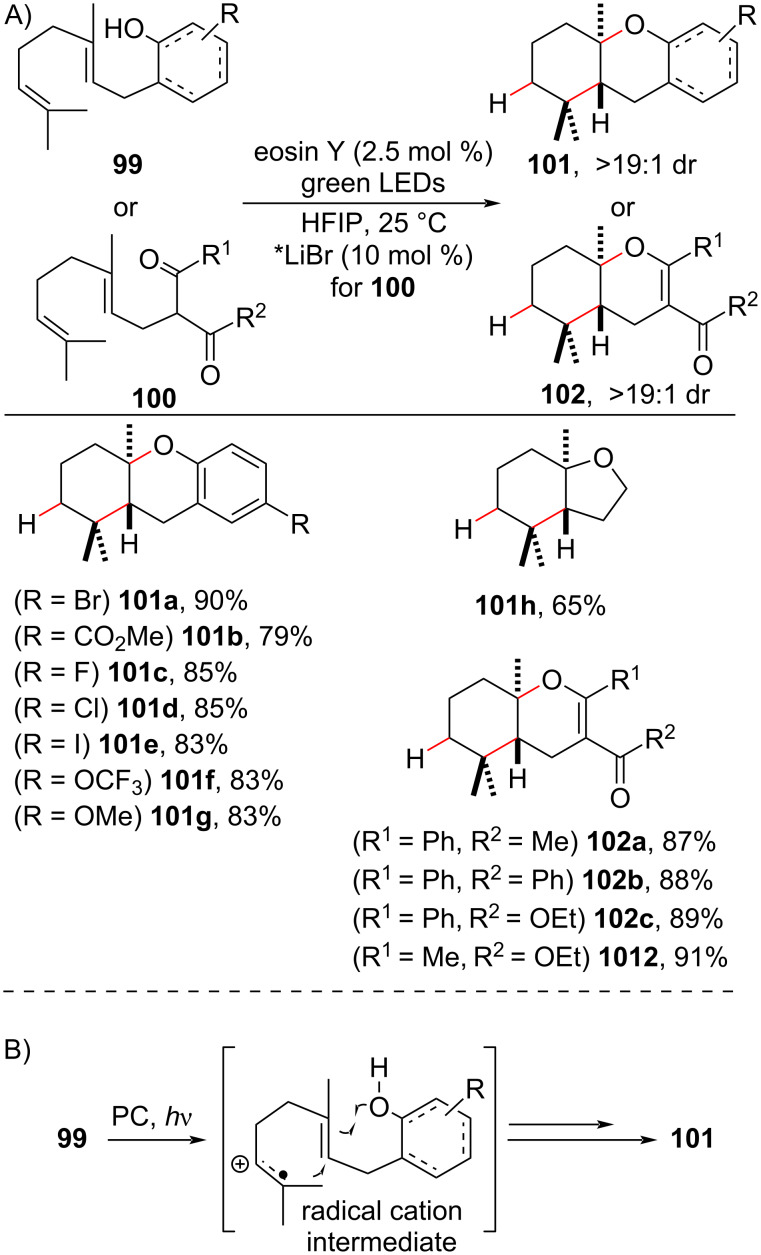
A) Selected examples of organophotocatalysis-mediated radical polyene cyclization via a PET process. B) Simplified view of the reaction mechanism (Luo (2015) [121]).

Under photoinduced electron transfer (PET) conditions, olefins generate cation radical species that are useful intermediates in polyene cyclization cascades [78,120]. Usually, the cation formed (see Scheme 38B) is trapped in an *anti*-Markovnikov fashion by a nucleophile, such as water or alcohol, present in the medium, while the radical engages in the cyclization cascade. Interestingly, in 2015, Luo reported that when a polyene **99** or **100** containing an acidic hydrogen in its structure (e.g.*,* a free hydroxy or enol) was subjected to a PET process, the radical cascade was accompanied by a hydrogen shift to give the formal hydroalkylated product **100** or **101** containing a quaternary carbon center (Scheme 38) [121]. Notably, the organic photocatalyst eosin Y was employed, and the cyclizations proceeded with excellent diastereoselectivity, usually higher than 19:1. When 1,3-ketocarbonyl substrates **100** were employed, the use of a weak Lewis acid (LiBr) was required to accomplish the cyclizations, and no reaction was observed in its absence. The authors highlighted the role of the solvent hexafluoro-2-propanol (HFIP) in the stabilization of the radical cation induced by PET and its assistance in the hydrogen shift process.

### Miscellaneous

#### Lewis acid catalysis in olefin hydroalkylation reactions

**Scheme 39:**
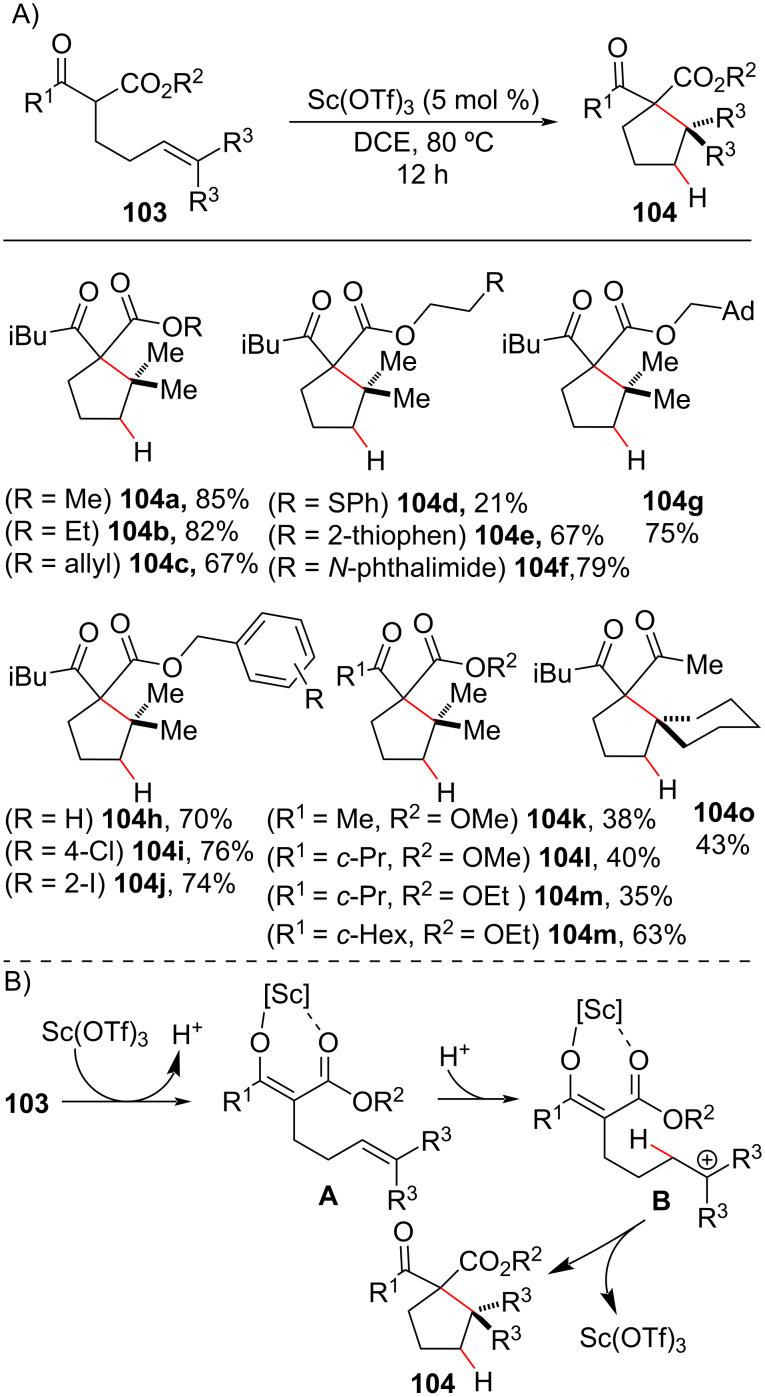
A) Sc(OTf)_3_-mediated carbocyclization approach for the synthesis of vicinal quaternary carbon centers. B) Proposed reaction mechanism (Schindler (2018) [122]).

The ability of a Lewis acid (LA) to activate 1,3-dicarbonyls through the formation of metallic enolates was recently explored by Schindler’s group for the intramolecular *tert*-alkylation of prenylated β-keto esters **103** (Scheme 39A) [122]. Cyclopentanes **104** containing two vicinal quaternary carbon centers were synthetized in yields of up to 90% under scandium triflate (Sc(OTf)_3_) catalysis. The mild conditions employed tolerated the presence of a diversity of functional groups in the ester moiety, including an allyl portion (Scheme 39, product **104c**) that could lead to competitive cyclization issues in other synthetic approaches. The reaction scope was limited to alkyl trisubstituted olefins. The authors proposed that the proton generated during scandium enolate **A** formation adds to the olefin double bond to generate a tertiary carbocation (**B**) that, after an intramolecular attack, affords cyclopentanes **104** (Scheme 39B).

**Scheme 40:**
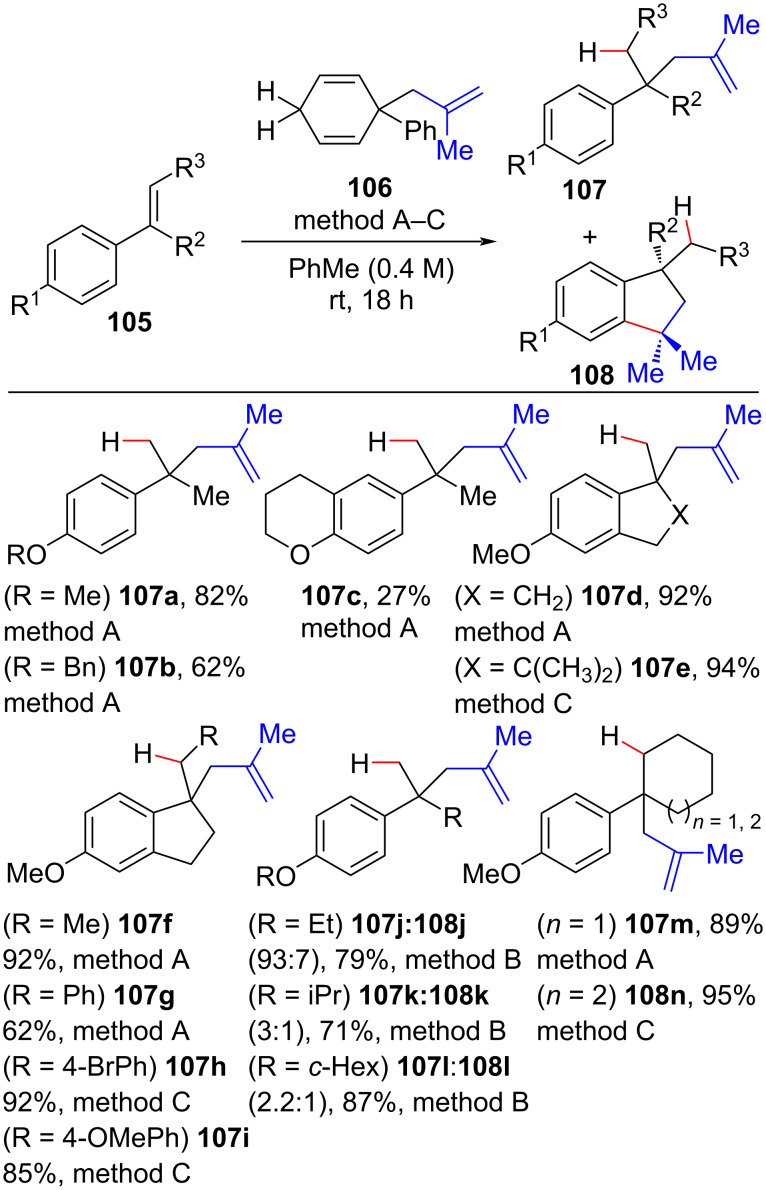
Scope of the Lewis acid-catalyzed methallylation of electron-rich styrenes. Method A: B(C_6_F_5_)_3_ (5.0 mol %), surrogate **106** (1.3 equiv). Method B: B(C_6_F_5_)_3_ (5.0 mol %), surrogate **106** (2.0 equiv). Method C: B(C_6_F_5_)_3_ (7.5 mol %), surrogate 10 (2.0 equiv) (Oestreich (2019) [123]).

A few examples exist of metal-free approaches for the generation of new C(sp^3^)–C(sp^3^) bonds at the cost of an unactivated olefin π-bond. In 2019, the Oestreich group successfully developed a metal-free methodology that allowed the hydromethallylation of styrenes using the electron-deficient Lewis acid B(C_6_F_5_)_3_ as a catalyst for the methallyl group transfer from a 1,4-cyclohexadiene methallyl surrogate **106** (Scheme 40) [123]. In general, a low catalyst load (5 mol %) and a slight excess of **106** (1.3 equiv) were employed at room temperature to synthetize the hydromethallylation products **107**. An evaluation of the reaction scope showed that *p*-methoxylated styrenes were the most suitable substrates and that changes in this moiety led to lower yields (**107b** and **107c**). Substitutions at the α-position of the olefin (R^2^) with more sterically demanding groups led to the observation of indane byproducts (**108j–l**). Substitutions at the terminal styrene olefin carbon (R^3^) were tolerable, although the presence of aromatic moieties (**105g–i**) necessitated an increase in the catalyst load and an excess of surrogate **106**.

**Scheme 41:**
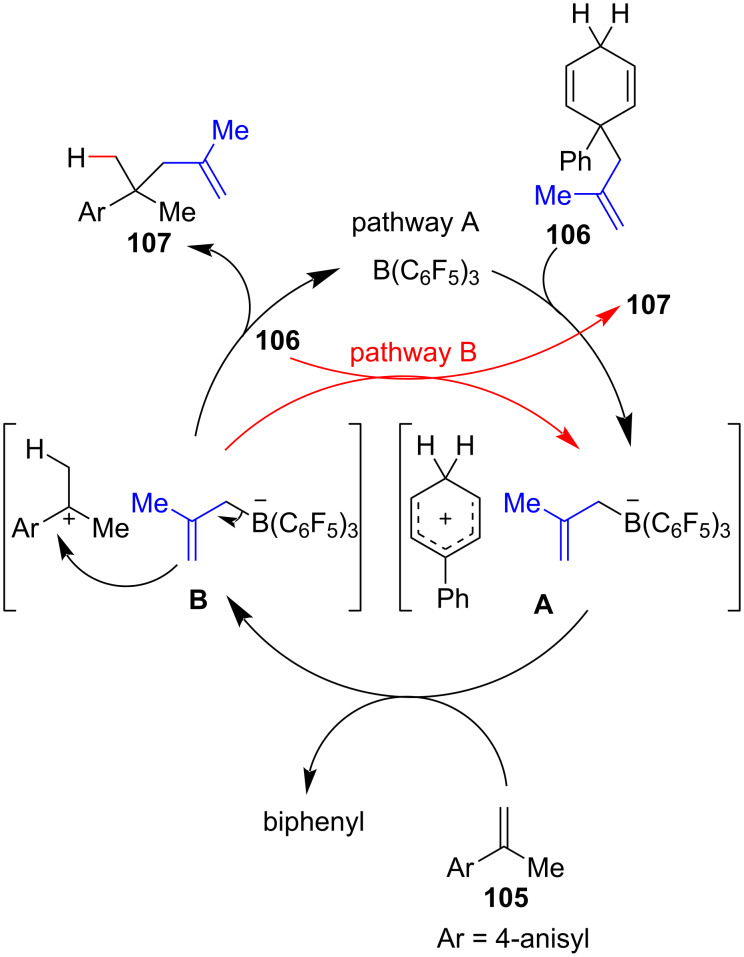
The proposed mechanism for styrene methallylation (Oestreich (2019) [123]).

The proposed mechanism relies on the generation of ionic complex **A** by the transfer of the methallyl group from surrogate **106** to the electron-deficient Lewis acid (Scheme 41). The Wheland intermediate that is generated then acts as a Brønsted acid, transferring a proton to the styrene (this step could explain the requirement for electron-rich styrenes) and leading to the formation of a new ionic complex **B** between the benzylic carbocation and the anionic borate species. Beyond this point, product **107** can be obtained by the methallyl group transfer by the borate species, thereby regenerating the Lewis acid catalyst, or by the direct transfer of the methallyl group from **106** to the benzylic carbocation in **B**.

## Conclusion

Since Widenhoefer [25] first reported the hydroalkylation of unactivated olefins using palladium catalysis in 2001, great advances in olefin functionalization have been achieved. Olefins display diverse reactivity patterns, including serving as electrophile partners in the presence of electrophilic transition-metal complexes or in atom transfer reactions or acting as nucleophile partners in hydrogen atom transfer reactions, and these patterns explain the continuous interest in exploring their chemistry. As this review demonstrates, olefin hydroalkylation reactions have been successfully developed for the construction of challenging quaternary carbon centers, leading to compounds with increased molecular complexity. The observed tendency in drug discovery programs toward the successful synthesis of compounds with more complex architecture than is traditionally obtained using the well-established transition metal C(*sp*^2^)–C(sp^2^) and C(sp*^2^*)–C(sp) cross-coupling reactions provides great encouragement for continuing to explore these nontraditional reactions.

## References

[1] Newman D J, Cragg G M (2016). J Nat Prod.

[2] Rodrigues T, Reker D, Schneider P, Schneider G (2016). Nat Chem.

[3] Dewick P M (2009). Medicinal Natural Products: A Biosynthetic Approach.

[4] Clardy J, Walsh C (2004). Nature.

[5] Long R, Huang J, Gong J, Yang Z (2015). Nat Prod Rep.

[6] Lovering F, Bikker J, Humblet C (2009). J Med Chem.

[7] Büschleb M, Dorich S, Hanessian S, Tao D, Schenthal K B, Overman L E (2016). Angew Chem, Int Ed.

[8] Tian L, Luo Y-C, Hu X-Q, Xu P-F (2016). Asian J Org Chem.

[9] Feng J, Holmes M, Krische M J (2017). Chem Rev.

[10] Li Y, Xu S (2018). Chem – Eur J.

[11] Hu P, Chi H M, DeBacker K C, Gong X, Keim J H, Hsu I T, Snyder S A (2019). Nature.

[12] Xu P-W, Yu J-S, Chen C, Cao Z-Y, Zhou F, Zhou J (2019). ACS Catal.

[13] Li C, Ragab S S, Liu G, Tang W (2020). Nat Prod Rep.

[14] Quasdorf K W, Overman L E (2014). Nature.

[15] Lovering F (2013). MedChemComm.

[16] Cox B, Zdorichenko V, Cox P B, Booker-Milburn K I, Paumier R, Elliott L D, Robertson-Ralph M, Bloomfield G (2020). ACS Med Chem Lett.

[17] James D H, Castor W M (2011). Styrene. Ullmann’s Encyclopedia of Industrial Chemistry.

[18] Dénès F, Pérez-Luna A, Chemla F (2010). Chem Rev.

[19] McDonald R I, Liu G, Stahl S S (2011). Chem Rev.

[20] Patil N T, Kavthe R D, Shinde V S (2012). Tetrahedron.

[21] Dong Z, Ren Z, Thompson S J, Xu Y, Dong G (2017). Chem Rev.

[22] O’Duill M L, Engle K M (2018). Synthesis.

[23] Smidt J, Hafner W, Jira R, Sedlmeier J, Sieber R, Rüttinger R, Kojer H (1959). Angew Chem.

[24] Jira R (2009). Angew Chem, Int Ed.

[25] Pei T, Widenhoefer R A (2001). J Am Chem Soc.

[26] Hegedus L S, Williams R E, McGuire M A, Hayashi T (1980). J Am Chem Soc.

[27] Hayashi T, Hegedus L S (1977). J Am Chem Soc.

[28] Hegedus L S, Darlington W H (1980). J Am Chem Soc.

[29] Qian H, Pei T, Widenhoefer R A (2005). Organometallics.

[30] Mecking S, Johnson L K, Wang L, Brookhart M (1998). J Am Chem Soc.

[31] Pei T, Widenhoefer R A (2002). Chem Commun.

[32] Wang X, Pei T, Han X, Widenhoefer R A (2003). Org Lett.

[33] Wang X, Widenhoefer R A (2004). Chem Commun.

[34] Liu C, Bender C F, Han X, Widenhoefer R A (2007). Chem Commun.

[35] Yang K S, Gurak J A, Liu Z, Engle K M (2016). J Am Chem Soc.

[36] Li Z, Brouwer C, He C (2008). Chem Rev.

[37] Chiarucci M, Bandini M (2013). Beilstein J Org Chem.

[38] Yao X, Li C-J (2004). J Am Chem Soc.

[39] Ranieri B, Escofet I, Echavarren A M (2015). Org Biomol Chem.

[40] Yao X, Li C-J (2005). J Org Chem.

[41] Zhou C-Y, Che C-M (2007). J Am Chem Soc.

[42] Fang W, Presset M, Guérinot A, Bour C, Bezzenine-Lafollée S, Gandon V (2014). Org Chem Front.

[43] Asao N, Aikawa H, Yamamoto Y (2004). J Am Chem Soc.

[44] Xiao Y-P, Liu X-Y, Che C-M (2011). Angew Chem, Int Ed.

[45] Xiao Y-P, Liu X-Y, Che C-M (2011). Beilstein J Org Chem.

[46] Kischel J, Michalik D, Zapf A, Beller M (2007). Chem – Asian J.

[47] Takeuchi R, Sagawa J, Fujii M (2019). Org Lett.

[48] Hartung J, Norton J R (2010). Catalysis Involving the H• Transfer Reactions of First-Row Transition Metals. Catalysis without Precious Metals.

[49] Gansäuer A, Shi L, Otte M, Huth I, Rosales A, Sancho-Sanz I, Padial N M, Oltra J E, Heinrich M, Gansäuer A (2012). Hydrogen Atom Donors: Recent Developments BT. Radicals in Synthesis III.

[50] Simonneau A, Oestreich M (2015). Angew Chem, Int Ed.

[51] Crossley S W M, Obradors C, Martinez R M, Shenvi R A (2016). Chem Rev.

[52] Nguyen K D, Park B Y, Luong T, Sato H, Garza V J, Krische M J (2016). Science.

[53] Lepori C, Hannedouche J (2017). Synthesis.

[54] Ai W, Zhong R, Liu X, Liu Q (2019). Chem Rev.

[55] Togo H, Togo H (2004). What Are Free Radicals?. Advanced Free Radical Reactions for Organic Synthesis.

[56] Isayama S, Mukaiyama T (1989). Chem Lett.

[57] Isayama S, Mukaiyama T (1989). Chem Lett.

[58] Mukaiyama T, Yamada T (1995). Bull Chem Soc Jpn.

[59] Okamoto T, Oka S (1984). J Org Chem.

[60] Okamoto T, Oka S (1984). J Chem Soc, Chem Commun.

[61] Okamoto T, Sasaki Y, Sasaki K, Oka S (1987). Bull Chem Soc Jpn.

[62] Waser J, Carreira E M (2004). J Am Chem Soc.

[63] Waser J, Carreira E M (2004). Angew Chem, Int Ed.

[64] Waser J, Nambu H, Carreira E M (2005). J Am Chem Soc.

[65] Waser J, González-Gómez J C, Nambu H, Huber P, Carreira E M (2005). Org Lett.

[66] Waser J, Gaspar B, Nambu H, Carreira E M (2006). J Am Chem Soc.

[67] Ishikawa H, Colby D A, Seto S, Va P, Tam A, Kakei H, Rayl T J, Hwang I, Boger D L (2009). J Am Chem Soc.

[68] Barker T J, Boger D L (2012). J Am Chem Soc.

[69] Leggans E K, Barker T J, Duncan K K, Boger D L (2012). Org Lett.

[70] Giedyk M, Goliszewska K, Gryko D (2015). Chem Soc Rev.

[71] Shey J, McGinley C M, McCauley K M, Dearth A S, Young B T, van der Donk W A (2002). J Org Chem.

[72] Crossley S W M, Barabé F, Shenvi R A (2014). J Am Chem Soc.

[73] Shevick S L, Wilson C V, Kotesova S, Kim D, Holland P L, Shenvi R A (2020). Chem Sci.

[74] Pitre S P, Weires N A, Overman L E (2019). J Am Chem Soc.

[75] Tao D J, Muuronen M, Slutskyy Y, Le A, Furche F, Overman L E (2016). Chem – Eur J.

[76] Hartung J, Pulling M E, Smith D M, Yang D X, Norton J R (2008). Tetrahedron.

[77] Choi J, Tang L, Norton J R (2007). J Am Chem Soc.

[78] Barrett A, Ma T-K, Mies T (2019). Synthesis.

[79] Lo J C, Yabe Y, Baran P S (2014). J Am Chem Soc.

[80] Lo J C, Kim D, Pan C-M, Edwards J T, Yabe Y, Gui J, Qin T, Gutiérrez S, Giacoboni J, Smith M W (2017). J Am Chem Soc.

[81] Kim D, Rahaman S M W, Mercado B Q, Poli R, Holland P L (2019). J Am Chem Soc.

[82] George D T, Kuenstner E J, Pronin S V (2015). J Am Chem Soc.

[83] Deng H, Cao W, Liu R, Zhang Y, Liu B (2017). Angew Chem, Int Ed.

[84] Godfrey N A, Schatz D J, Pronin S V (2018). J Am Chem Soc.

[85] Lu Z, Zhang X, Guo Z, Chen Y, Mu T, Li A (2018). J Am Chem Soc.

[86] Huang J-K, Yang Lauderdale T-L, Lin C-C, Shia K-S (2018). J Org Chem.

[87] Cao W, Deng H, Sun Y, Liu B, Qin S (2018). Chem – Eur J.

[88] Nagasawa S, Jones K E, Sarpong R (2019). J Org Chem.

[89] Dokli I, Pohl R, Klepetářová B, Jahn U (2019). Chem Commun.

[90] Zeng X, Shukla V, Boger D L (2020). J Org Chem.

[91] Shen Y, Qi J, Mao Z, Cui S (2016). Org Lett.

[92] Qi J, Zheng J, Cui S (2018). Org Lett.

[93] Qi J, Zheng J, Cui S (2018). Org Chem Front.

[94] Qi J, Tang H, Chen C, Cui S, Xu G (2019). Org Chem Front.

[95] Dao H T, Li C, Michaudel Q, Maxwell B D, Baran P S (2015). J Am Chem Soc.

[96] Saladrigas M, Bosch C, Saborit G V, Bonjoch J, Bradshaw B (2018). Angew Chem, Int Ed.

[97] Saladrigas M, Loren G, Bonjoch J, Bradshaw B (2018). ACS Catal.

[98] Saladrigas M, Bonjoch J, Bradshaw B (2020). Org Lett.

[99] Vrubliauskas D, Vanderwal C D (2020). Angew Chem, Int Ed.

[100] Geist E, Kirschning A, Schmidt T (2014). Nat Prod Rep.

[101] Ishiyama T, Abe S, Miyaura N, Suzuki A (1992). Chem Lett.

[102] Jones G D, Martin J L, McFarland C, Allen O R, Hall R E, Haley A D, Brandon R J, Konovalova T, Desrochers P J, Pulay P (2006). J Am Chem Soc.

[103] Lin X, Phillips D L (2008). J Org Chem.

[104] Schley N D, Fu G C (2014). J Am Chem Soc.

[105] Choi J, Fu G C (2017). Science.

[106] Fu G C (2017). ACS Cent Sci.

[107] Lu X, Xiao B, Zhang Z, Gong T, Su W, Yi J, Fu Y, Liu L (2016). Nat Commun.

[108] Wang Z, Yin H, Fu G C (2018). Nature.

[109] Green S A, Huffman T R, McCourt R O, van der Puyl V, Shenvi R A (2019). J Am Chem Soc.

[110] Green S A, Vásquez-Céspedes S, Shenvi R A (2018). J Am Chem Soc.

[111] Stolle A, Ondruschka B, Hopf H (2009). Helv Chim Acta.

[112] Sumino S, Fusano A, Ryu I (2013). Org Lett.

[113] Nakajima M, Lefebvre Q, Rueping M (2014). Chem Commun.

[114] Miura T, Funakoshi Y, Nakahashi J, Moriyama D, Murakami M (2018). Angew Chem, Int Ed.

[115] Qin Q, Wang W, Zhang C, Song S, Jiao N (2019). Chem Commun.

[116] Lopp J M, Schmidt V A (2019). Org Lett.

[117] Curran D P, Chen M H, Spletzer E, Seong C M, Chang C T (1989). J Am Chem Soc.

[118] Povie G, Suravarapu S R, Bircher M P, Mojzes M M, Rieder S, Renaud P (2018). Sci Adv.

[119] Tian Y, Ling A, Fang R, Tan R X, Liu Z-Q (2018). Green Chem.

[120] Heinemann C, Demuth M (1999). J Am Chem Soc.

[121] Yang Z, Li H, Zhang L, Zhang M-T, Cheng J-P, Luo S (2015). Chem – Eur J.

[122] McAtee C C, Ellinwood D C, McAtee R C, Schindler C S (2018). Tetrahedron.

[123] Walker J C L, Oestreich M (2019). Angew Chem, Int Ed.

